# Measuring co-constructive collaboration between general and special education teachers in inclusive schools—development and validation of two short questionnaires

**DOI:** 10.3389/fpsyg.2025.1535727

**Published:** 2025-06-17

**Authors:** Jacquelin Kluge, Benjamin A. Korman, Janine Schledjewski, Michael Grosche

**Affiliations:** ^1^University of Wuppertal, Wuppertal, Germany; ^2^Leibniz Institute for Educational Trajectories, Bamberg, Germany

**Keywords:** co-constructive collaboration, general education teachers, inclusive education, special education teachers, questionnaire development, validation

## Abstract

**Introduction:**

Collaboration between general and special education teachers is important for the successful implementation of inclusive education. In this article, we discuss three forms of collaboration, with a particular focus on co-constructive collaboration as the most intensive and promising form for implementing inclusive education. Based on the theoretical framework of co-constructive collaboration, we validate two short questionnaires—in German as well as in English—for measuring co-constructive collaboration between general and special education teachers.

**Method:**

Across six studies involving a total of 2.332 general and special education teachers, we conducted both exploratory and confirmatory factor analyses, examined convergent validity, and investigated whether the measurement model of our scales is invariant between (1) general and special education teachers, (2) primary and secondary school teachers, and (3) German- and English-speaking teachers.

**Results:**

The results reveal two reliable instruments: (1) one that assesses a comprehensive view of co-constructive collaboration, encompassing requirements, co-constructive activities, and outcomes, and (2) one that specifically measures teachers’ commitment to collaboration and iterative revision as a distinct co-constructive activity. The subscales largely correlate with related constructs, such as attitudes towards inclusion, confirming convergent validity. While measurement invariance is established for general and special education teachers, the results for the comparison between primary and secondary school teachers as well as between German- and English-speaking teachers are, with the exception of the latter group in the first instrument, less satisfactory. However, the respective factor structures of the individual groups are satisfactory.

**Discussion:**

The findings demonstrate the reliability and validity of the newly developed instruments for measuring core-aspects of co-constructive collaboration between general and special education teachers in German- and English-speaking inclusive schools, supporting cross-cultural research in inclusive education. Study limitations, such as the partial lack of measurement invariance, are also discussed.

## 1 Introduction

Internationally, collaboration between teachers is considered important for the positive development of students, teachers, and schools in education in general (e.g., Hargreaves, 2019; Joney et al., 2019; Vangrieken et al., 2015) as well as in inclusive education (e.g., Holmqvist and Lelinge, 2021). In particular, collaboration between general education teachers (GETs) and special education teachers (SETs) is regarded as a key factor for the successful implementation of inclusive education (Finkelstein et al., 2021; Hoppey and McLeskey, 2014; Jones and Winters, 2024; Neumann and Lütje-Klose, 2021; Mouchritsa et al., 2021). It is assumed that the development of inclusive schools, as well as classrooms, in which the diverse needs of students are addressed, cannot be successfully managed by a single teacher but instead requires the bringing together of different expertise (Grosche et al., 2020; Jones and Winters, 2024).

In schools, however, it is not always clear how collaboration should be organised. For example, in Germany, where the present study was primarily conducted, most GETs and SETs undergo separate university training programmes, historically designed to prepare them for different school types. With the development of inclusive education, SETs are no longer confined to special schools but increasingly work alongside GETs in inclusive settings. Since the individual federal states in Germany decide on their education systems, there are differences in policies and structures across regions. Overall, there is a lack of regulations regarding the tasks and responsibilities of SETs and consequently also the structure of collaboration between GETs and SETs. As a result, teachers often have to take responsibility for their roles and tasks and for shaping their collaboration themselves (Dietze et al., 2023). Accordingly, how GETs and SETs collaborate in schools varies greatly, with collaboration often being weak or not strongly developed (Neumann and Lütje-Klose, 2021). The ambiguous role of SETs, as well as the variation and lack of collaboration, have also been observed in other European countries and the United States (Björn et al., 2016; Ciletti et al., 2025; Mouchritsa et al., 2021), making this a challenge faced by teachers across the globe.

Collaboration is also a rather diffuse concept in the educational literature. There is no generally accepted definition of “collaboration” (Kelchtermans, 2006). Rather, collaboration represents a diverse and complex construct (Drossel et al., 2019). Differences relate, for example, to the people involved, the form or intensity, the content, the context, the function or objective, or the underlying theoretical construct (Drossel et al., 2019; Kelchtermans, 2006). This complexity is also evident in the different questionnaires used to measure collaboration in educational research (e.g., Decuyper et al., 2023; DeLuca et al., 2023; Honingh and Hooge, 2014; Johari et al., 2022; Mora-Ruano et al., 2018; Zhang and Zheng, 2020). However, not all studies provide (sufficient) information on the development and psychometric quality of the instruments (cf. Flake et al., 2017 or Hinkin et al., 1997 for necessary steps in construct validation). Moreover, the questionnaires are sometimes based on broader theoretical or empirical considerations rather than a concrete theoretical framework. This can lead to inconsistencies, such as the conflation of characteristics regarding the relationship between teachers and their actual collaborative activities (cf. differences between collegiality and collaboration; Kelchtermans, 2006). In order to properly understand and evaluate the collaboration of teachers in schools, it is necessary to establish theoretical frameworks and psychometrically sound measures (Grosche and Moser Opitz, 2023; Joney et al., 2019).

One influential model of collaboration in Germany, which has significantly impacted research on collaboration (e.g., Webs and Holtappels, 2018; Drossel et al., 2019; Pozas and Letzel-Alt, 2023; Muckenthaler et al., 2020; Wiedebusch et al., 2022), comes from Gräsel et al. (2006). This model captures teaching-related collaboration, which includes collaboration both in and outside the classroom. It has been applied in contexts involving interdisciplinary collaboration between GETs and SETs, as well as multiprofessional collaboration among teachers and other professionals in schools (Neumann and Lütje-Klose, 2021). In their model, Gräsel et al. (2006) differentiate between three forms of collaboration: (1) exchange, (2) division of work, and (3) co-constructive collaboration. These forms differ in their intensity and function. In recent work by Grosche et al. (2020), the authors present a revised theoretical framework for co-constructive collaboration between GETs and SETs which is seen as the most beneficial form of collaboration for inclusive education.

The aim of this paper is twofold: First, we discuss the three forms of collaboration differentiated by Gräsel et al. (2006) and their functions in the context of inclusion, with a particular focus on the theoretical framework of co-constructive collaboration by Grosche et al. (2020) and its promises for inclusive education. Second, we present the theory-based development and validation of two short questionnaires designed to measure co-constructive collaboration between GETs and SETs in inclusive schools. Importantly, we test our scales in both German- and English-speaking samples to enable their use in different national and demographic contexts. Thus, our work represents a first step towards cross-cultural validation and provides an avenue for future cross-cultural studies on teacher collaboration in inclusive schools.

### 1.1 Exchange, division of work, and co-constructive collaboration in the context of inclusion

Gräsel et al. (2006) define collaboration based on the definition proposed by Spieß (2004). Consequently, “collaboration is characterised by the reference to others, to common goals or tasks, it is intentional, communicative and requires trust. It presupposes a certain degree of autonomy and is committed to the norm of reciprocity” (Spieß, 2004, p. 199, translated by the authors). In their model, Gräsel et al. (2006) differentiate three forms of collaboration, which differ in their intensity and function (Grosche et al., 2020; Gräsel et al., 2006). The first form of collaboration is the *exchange* of information and materials. The second form is *division of work* (Webs and Holtappels, 2018), which has also been referred to in the literature as “synchronization” (Drossel et al., 2019; Pozas and Letzel-Alt, 2023) or “coordination/shared work” (Neumann and Lütje-Klose, 2021). Teachers divide tasks, work on them independently, and then combine their results to achieve a common task or goal (Grosche et al., 2020). Both forms are regarded as less intensive and particularly suited to routine tasks that can, for the most part, be completed independently. In the context of inclusive education, exchange and division of work may occur when GETs and SETs exchange information about individual students or agree on a shared teaching objective, which is then planned and implemented separately for different groups of students (Grosche et al., 2020).

The third and most intensive form of collaboration is *co-constructive collaboration* (CCC). A key characteristic of CCC, and what sets it apart from the other two forms, is the joint development of strategies for dealing with complex educational challenges, such as the implementation of inclusive education. CCC is evident, for example, when GETs and SETs collaboratively plan and conduct a lesson for *all* students, ensuring that everyone is fully integrated, rather than teaching individual students or groups of students separately (Grosche et al., 2020).

In the following, we focus on CCC. As mentioned above, it is assumed that this form is most effective for implementing inclusive education (Grosche et al., 2020). One of the reasons for this assumption is that inclusive education requires the bringing together of different expertise, which cannot be achieved through exchange or division of work alone. Rather, collaborative processes of negotiation, planning, and reflection are needed in order to successfully implement inclusive education (Grosche et al., 2020).

### 1.2 Theoretical framework of co-constructive collaboration

**Figure 1:**
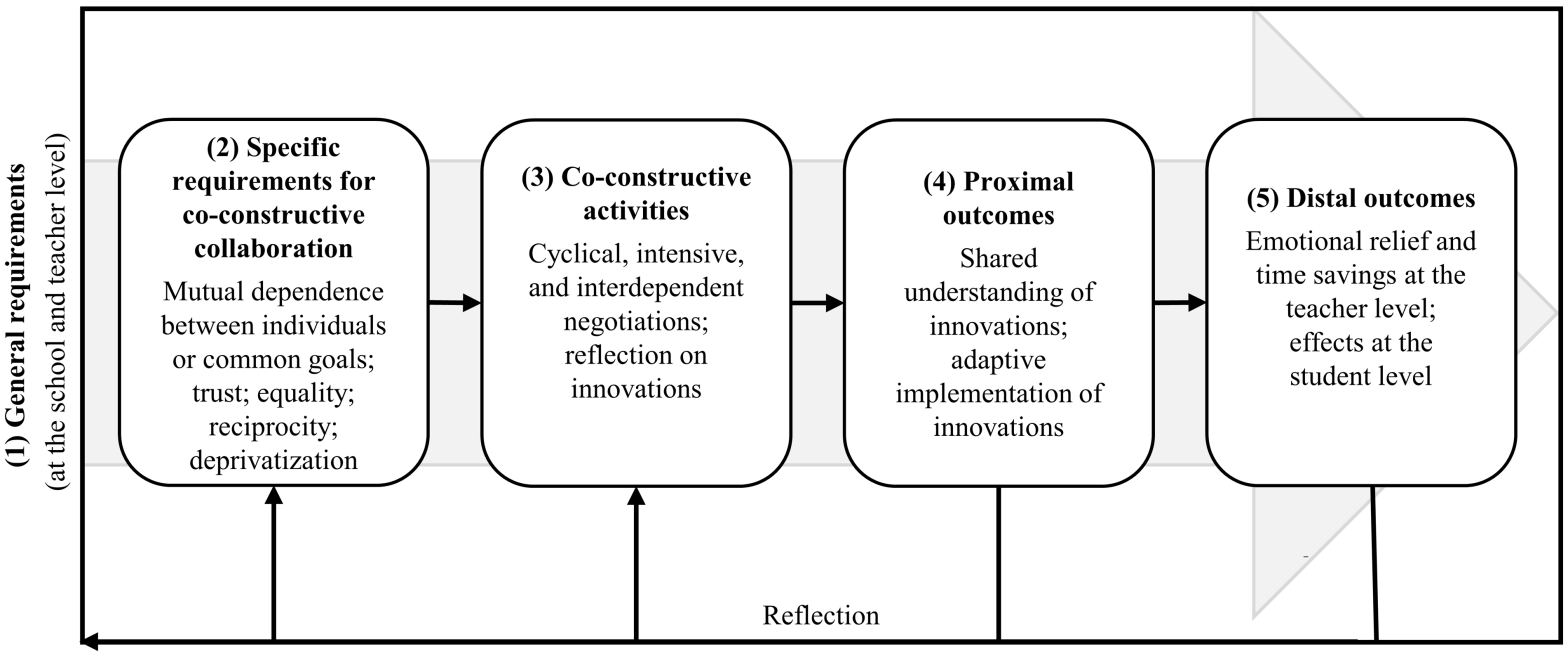
Framework of co-constructive collaboration (adapted from Grosche et al., 2020).

Grosche et al. (2020) developed a theoretical framework for CCC that serves as the basis for the two questionnaires developed and evaluated in this study. Figure 1 illustrates the framework and its five fundamental dimensions: (1) general requirements, (2) specific requirements for CCC, (3) co-constructive activities, (4) proximal outcomes, and (5) distal outcomes.

(1) General requirements are important for all forms of collaboration. On the one hand, this concerns aspects at the teacher level, such as a positive attitude towards and positive experiences with collaboration (e.g., DeLuca et al., 2023; Vangrieken et al., 2015), mutual sympathy, or respect (e.g., Paulsrud and Nilholm, 2023; Scruggs et al., 2007). On the other hand, aspects at the school level are important for collaboration. These include structural conditions, such as the availability of time slots for collaboration (e.g., Paulsrud and Nilholm, 2023; Vangrieken et al., 2015; Webs and Holtappels, 2018) as well as cultural conditions, including school leadership that supports collaboration among teachers (e.g., Paulsrud and Nilholm, 2023; Vangrieken et al., 2015; Wiedebusch et al., 2022; Zhang and Zheng, 2020).

According to the framework, (2) specific requirements include, first and foremost, goal interdependence, where teachers either share a common goal or have individual goals that are intertwined. The existence of a common goal or intertwined goals enables teachers to perceive the necessity for joint and intensive processes of negotiation and reflection (Grosche et al., 2020). Furthermore, it is assumed that trust, reciprocity, equal and symmetric communication (equality), and the willingness to deprivatize one’s own teaching are required. Other theoretical frameworks and reviews similarly highlight the importance of these aspects for teacher collaboration (e.g., Mouchritsa et al., 2021; Neumann and Lütje-Klose, 2021; Scruggs et al., 2007; Vangrieken et al., 2015). In the framework, they are seen as specific to CCC, because it is assumed that they are required to a much lesser extent for the other two forms of collaboration (Grosche et al., 2020). However, based on the few existing studies, which found different correlations between individual requirements and the three different forms of collaboration (e.g., Webs and Holtappels, 2018), no systematic statements can be made to date concerning conditions specific to different forms of collaboration.

The model is centred upon (3) co-constructive activities that represent the actual collaboration between teachers. These activities define CCC as “joint, cyclical, intensive, and interdependent processes of negotiation and reflection on innovations and their concretization” (Grosche et al., 2020, p. 467, translated by the authors). When individual goals are present, the first step in these co-constructive activities would be to negotiate a common goal (Fussangel et al., 2023). In practice, however, it is evident that teachers collaborate less co-constructively with one another, opting rather for the forms of exchange and division of work (e.g., Pozas and Letzel-Alt, 2023; Webs and Holtappels, 2018). This is consistent with the finding reported in the introduction that intensive collaboration is implemented less frequently.

The last two dimensions of the model represent outcomes of CCC which are divided into (4) proximal outcomes and (5) distal outcomes (Grosche et al., 2020). Proximal outcomes are those that result directly from co-constructive activities and include a shared understanding of innovation (e.g., inclusive education) and the adaptive implementation of this innovation in the classroom (Grosche et al., 2020). Moreover, it is assumed that co-constructive activities can lead to shared responsibility among teachers and their overcoming of traditional roles and responsibilities (Grosche et al., 2020), both of which are considered important for successful inclusive education (Hoppey and McLeskey, 2014; Neumann and Lütje-Klose, 2021). The overcoming of traditional roles implies that SETs are no longer solely responsible for students with special educational needs and GETs for students without special educational needs. Distal outcomes lie outside of the co-constructive activities and are expected at the student level, such as improved learning, and at the teacher level, such as emotional relief or time saved (Grosche et al., 2020). These outcomes may be achieved, for example, when strategies for individually supporting student learning are successfully implemented in the classroom, leading to improved learning outcomes for students.

To date, studies on the outcomes of collaboration, including outcomes for the specific forms of exchange, division of work and CCC are rare (Grosche and Moser Opitz, 2023; Joney et al., 2019). That being said, two of the few existing studies investigating these different forms of collaboration indicate that CCC positively correlates with differentiated teaching, whereas the exchange form of collaboration does not (Pozas and Letzel-Alt, 2023; Webs and Holtappels, 2018). Additionally, preliminary evidence suggests that individual co-constructive activities, such as negotiating differences of opinion, may foster a shared sense of responsibility for all students among teachers and a more inclusive understanding of their role as teachers (Kluge and Grosche, 2021). However, co-constructive activities do not appear to lead to greater shared responsibility for different school tasks between GETs and SETs (Kluge and Grosche, 2021; Kluge et al., 2024). When not exclusively focusing on collaboration in the forms of exchange, division of work, or CCC, studies indicate that collaboration is associated with positive outcomes (e.g., King-Sears et al., 2021; Lochner et al., 2019; Paulsrud and Nilholm, 2023), including improved learning outcomes for students with and without special educational needs (Jones and Winters, 2024). Nevertheless, it should be noted that collaboration among teachers can also be associated with undesirable outcomes, such as students with special educational needs having less peer-interaction when a second teacher is present in the classroom (Spörer et al., 2021).

As shown in Figure 1, CCC is a cyclical framework. Teachers repeatedly engage in processes of negotiation and reflection. Moreover, the outcomes of CCC are expected to affect later co-constructive activities and requirements (Grosche et al., 2020). For example, teachers who experience successful collaboration (i.e., via improved learning outcomes of students) will likely have a more positive attitude towards future collaboration.

Overall, the assumptions of the CCC framework align with key elements emphasized in other theoretical models and studies on teacher collaboration. However, while the framework offers a comprehensive theoretical structure, its assumptions have so far only been partially supported by empirical research. The specific conditions, mechanisms, and outcomes of CCC still require systematic empirical investigation. This presupposes the availability of valid measurement instruments.

### 1.3 The present study

In this paper, we develop and evaluate two short form questionnaires to measure CCC between GETs and SETs. The items of these questionnaires were developed in accordance with the theory on CCC (Grosche et al., 2020) and, thus, address requirements for (co-constructive) collaboration, activities, and outcomes. Importantly, we focused solely on teacher-related aspects of collaboration while excluding requirements related to school conditions and distal outcomes at the student level. We chose this approach because our primary objective was to develop a questionnaire that specifically captures the dynamics and interactions between teachers, as these are directly within their control. For both of our questionnaires, we asked the following research questions:

1. Does the questionnaire adequately measure the main dimensions of the theory? Specifically, can a substantive and reliable factor structure be identified?
2. Does the measure of CCC demonstrate convergent construct validity?
3. Is the measurement model invariant between (1) GETs and SETs, (2) primary and secondary school teachers, and (3) German- and English-speaking teachers?

## 2 Method

### 2.1 Samples

The development of the questionnaires is based on six studies. The first four studies (Studies 1a to 1d) are drawn from four measurement points within the two projects “Inclusion in secondary schools in Germany” (INSIDE) and “Inclusion and transitions after secondary school in Germany” (INSIDE II). These projects involved longitudinal research examining the implementation of inclusive education in secondary schools in Germany (Gresch et al., in print). Thereby, teachers from 14 different federal states in Germany (with the exception of Berlin and Brandenburg, where grades 5 and 6 are connected to elementary school) were surveyed using paper-pencil. The remaining two studies (Studies 2 and 3) were conducted separately by the authors and administered online using the survey programme Lime Survey. In all studies, participants provided their informed consent to participate, either through submitting the questionnaire or through confirmation within the online survey platform, prior to starting the study.

In S*tudy 1a* (INSIDE, measurement point 1, spring 2019), *N* = 1,019 6th grade teachers participated. Eight teachers were excluded from the analyses, as they did not answer any of the items concerning CCC. Of the final *n* = 1,011 teachers, 825 worked as GETs and 186 as SETs. 76.7% were female, with the proportion of female teachers being slightly higher (*χ*^2^(1) = 6.309, *p* = 0.012) among SETs (83.9%) than among GETs (75.0%). The mean age of all participants was 42.54 (*SD* = 11.02) years old and they had an average of 13.75 (*SD* = 10.82) years of teaching experience. GETs and SETs did not differ in age (*t*(1,000) = 1.156, *p* = 0.248) or years of teaching experience (*t*(953) = 1.190, *p* = 0.234).

In *Study 1b* (INSIDE, measurement point 2, spring 2020), *N* = 500 7th grade teachers participated. Seven teachers were excluded from the analyses, as they did not answer any of the items concerning CCC. Of the final teachers (*n* = 493), 406 worked as GETs and 87 as SETs. 73.8% were female. The mean age of all participants was 44.21 (*SD* = 10.93) years old and they had an average of 15.16 (*SD* = 11.19) years of teaching experience. GETs and SETs did not differ in gender (*χ*^2^(1) = 1.824, *p* = 0.177), age (*t*(482) = 1.378, *p* = 0.169) or years of teaching experience (*t*(468) = 1.578, *p* = 0.115). Two-hundred-eighty-eight teachers had already participated in Study 1a.

In *Study 1c* (INSIDE II, measurement point 3, spring 2022), *N* = 293 9th grade teachers participated. Two-hundred-thirty-one worked as GETs and 43 as SETs. 70.8% were female. The mean age of all participants was 45.18 (*SD* = 10.52) years old and they had an average of 15.73 (*SD* = 10.86) years of teaching experience. GETs and SETs did not differ in gender (*χ*^2^(1) = 0.194, *p* = 0.659), age (*t*(286) = 1.389, *p* = 0.166) or years of teaching experience (*t*(223) = 0.507, *p* = 0.613). Seventy-three teachers had already participated in both previous studies, 60 only in one of the previous two studies, and 160 were participating for the first time.

In *Study 1d* (INSIDE II, measurement point 4, spring 2023), *N* = 198 10th grade teachers participated. Three teachers were excluded from the analyses, as they did not answer any of the items concerning CCC. Of the final *n* = 195 teachers, 171 worked as GETs and 24 as SETs. 65.6% were female. The mean age of all participants was 45.91 (*SD* = 10.08) years old and they had an average of 16.56 (*SD* = 10.86) years of teaching experience. GETs and SETs did not differ in gender (*χ*^2^(1) = 0.072, *p* = 0.788), age (*t*(192) = −0.149, *p* = 0.881) or years of teaching experience (*t*(110) = −1.026, *p* = 0.307). Thirty-eight teachers had already participated in all three previous studies, 32 in two of the three previous studies, 63 in one of the three previous studies, and 62 were participating for the first time.

*Study 2* was conducted with master students, who assisted in recruiting teachers as part of a research seminar at the University of Wuppertal during the summer of 2023. *N* = 413 teachers (89.6% female) from primary schools (in grades 1–4) in North Rhine-Westphalia, Germany, filled out a questionnaire concerning their CCC. 322 worked as GETs and 91 as SETs. On average, participants were 43.08 (*SD* = 10.19) years old and they had an average of 14.56 (*SD* = 9.60) years of teaching experience. GETs and SETs did not differ in gender (*χ*^2^(1) = 0.159, *p* = 0.690), age (*t*(410) = 0.216, *p* = 0.830) or years of teaching experience (*t*(407) = 0.030, *p* = 0.976).

*Study 3* was conducted in autumn 2024 using the online platform Prolific (Palan and Schitter, 2018), a valuable source of reliable study participants (Douglas et al., 2023; Peer et al., 2017). Prolific maintains high data quality by regularly vetting its users and employing algorithms to detect and remove bots (Bradley, 2018). *N* = 481 teachers from elementary, middle, high schools, and colleges in the United States participated in this study.^1^ Three-hundred-eighty-eight worked as GETs and 93 as SETs. 70.7% were female, with the proportion of female teachers being slightly higher (*χ*^2^(1) = 4.135, *p* = 0.042) among SETs (80.7%) than among GETs (68.3%). The mean age was 39.79 (*SD* = 10.05) years old and they had an average of 12.14 (*SD* = 8.54) years of teaching experience. GETs and SETs did not differ in age (*t*(479) = −0.843, *p* = 0.400) or years of teaching experience (*t*(479) = 0.666, *p* = 0.505).

### 2.2 Measures

The first short questionnaire was developed as part of the INSIDE project. Its 17 items were pretested in two stages: first, through 13 qualitative interviews with German teachers, and then through a quantitative pre-test involving 181 teachers and 133 university students studying to become teachers in Germany. Five items that were either difficult to comprehend or ambiguous were excluded. Two items addressing the responsibilities of teachers for students as well as the equality between teachers were added retrospectively, resulting in a final set of 14 items. The second questionnaire also consists of 14 items. These were items initially from the long-version of the CCC questionnaire (Fussangel et al., 2023). They were also pretested in qualitative interviews with six German teachers. Both questionnaires measure respondents’ agreement with various statements concerning CCC using a four-point scale (1 = “strongly disagree,” 4 = “strongly agree”).

To test the construct validity of our proposed measures, we additionally measured four constructs considered important in the context of inclusion and collaboration. These included:

(1) Attitudes towards inclusion, which were measured using the short form of the Multi-Profession Scale for Attitudes to an Inclusive School System (*k* = 6, Bruns et al., 2023; Lüke and Grosche, 2020). Responses were rated on a four-point scale (1 = “strongly disagree,” 4 = “strongly agree”).
(2) Teacher self-efficacy: For the German-speaking samples, a scale measuring self-efficacy in relation to inclusive teaching based on Bosse and Spörer (2014) was used (*k* = 7). For the English-speaking sample, the subscale on collaboration of the short form of Teacher Efficacy for Inclusive Practice Scale was used (*k* = 3, Sahli Lozano et al., 2023). Both scales utilised a four-point rating scale (1 = “strongly disagree,” 4 = “strongly agree”).
(3) Teacher responsibility for students’ learning and student-teacher-relationship (for both subscales *k* = 3), which was measured using a questionnaire based on Lauermann and Karabenick (2013). Responses were rated on a four-point scale (1 = “not at all responsible,” 4 = “completely responsible”).
(4) Co-teaching, which was measured through six different co-teaching forms: ‘one teach, one observe,’ ‘one teach, one assist,’ ‘alternative teaching,’ ‘parallel teaching,’ ‘station teaching’, and ‘team teaching’ based on Friend et al. (2010) and Schledjewski et al. (2021). Co-teaching was assessed by asking participants about the frequency with which they implemented six different co-teaching forms, using a four-point scale ranging from 1 = “never” to 4 = “several times per week.” Each co-teaching form was analysed individually. It is important to note that the sample sizes for the co-teaching measures are smaller, as only teachers who taught classes with another teacher were surveyed. In some instances, multiple responses on co-teaching from the same teacher were available in Studies 1a and 1b, as teachers may have completed the questionnaire for different classes or subjects (e.g., German or mathematics). In these instances, teachers’ responses were aggregated at the individual level for analysis.

### 2.3 Procedure and statistical analyses

The development and evaluation of the two short form questionnaires to measure CCC between GETs and SETs was conducted in four steps that were applied to both questionnaires. In total, six studies were involved. Table 1 provides an overview of the steps, statistical analyses, and studies involved.

**Table 1:** Steps, statistical analyses, and studies involved.

Steps	Questionnaire 1	Questionnaire 2
1 Item analysis	Study 1a	Study 1b
2 Explorative and confirmatory factor analysis		
a) Split half: EFA and CFA	Study 1a	Study 1b
b) Further CFAs	Studies 1c, 1d, 2, 3	Studies 1c, 1d, 2, 3
3 Measurement invariance		
a) General and special education teachers	Study 1a	Study 1b
b) Primary and secondary school teachers	Studies 1a and 2	Studies 1b and 2
c) German- and English-speaking teachers	Studies 1a and 3	Studies 1b and 3
4 Convergent validity		
a) German version of the questionnaire	Study 1a	Study 1b
b) English version of the questionnaire	Study 3	Study 3

Studies 1a–1d were part of the INSIDE project, in which secondary school teachers from Germany were surveyed. Study 2 was conducted with primary school teachers located in Germany, and Study 3 with teachers located in the USA.

In the first step, an initial item analysis was performed to assess skewness, kurtosis, and item difficulty. In order to avoid items with unfavourable distribution and floor or ceiling effects, items with skew > |2|, kurtosis > |7| (Ryu, 2011), or an item difficulty <0.20 or >0.80 were excluded. For these analyses, we used data from Study 1a, where the first questionnaire was initially used, and data from Study 1b, where the second questionnaire was initially used.

In the second step, the data set (again comprising data from Study 1a for questionnaire 1 and from Study 1b for questionnaire 2) was randomly split into a training data set and a test data set. This was done in order to first identify the factor structure and then validate it on an independent sample (Anderson and Gerbing, 1988). After assessing the suitability of the training data set using the Kaiser-Meyer-Olkin test (KMO, with a value < 0.50 considered unacceptable, Kaiser, 1974) and Bartlett’s Test of Sphericity (Bartlett, 1937), an exploratory factor analysis (EFA) with oblique rotation was conducted. The analysis employed listwise deletion and maximum likelihood (ML) estimation. Items were considered for removal if they had cross-loadings > 0.32 or factor loadings < 0.50 (Tabachnick and Fidell, 2007). Additionally, communalities were evaluated, using a cut-off of 0.35. This threshold was chosen based on the existing recommendations, with cut-offs varying from 0.20 (Child, 2006) to 0.50 (Hair et al., 2010). Model fit was assessed using the criteria recommended by Hu and Bentler (1999): CFI and TLI > 0.95, RMSEA < 0.06, and SRMR < 0.08. The identified factor structure was then independently tested on the test data set using confirmatory factor analysis (CFA). An ML estimator was used along with full information maximum likelihood (FIML) for estimating missing values. The internal consistency reliability was assessed using McDonald’s Omega (McDonald, 1999). The items of the final model were collected in later time points (Studies 1c, 1d, 2, and 3). We used this data to conduct further CFAs.

In the third step, convergent construct validity was evaluated by examining correlations with related constructs: (1) attitudes towards inclusion, (2) self-efficacy, (3) teachers’ responsibilities for student achievement and student-teacher relationships, and (4) six different forms of co-teaching. Construct validity was tested for both the German (data from Study 1a for questionnaire 1 and Study 1b for questionnaire 2) and English versions (data from Study 3) of the questionnaires.

In the fourth step, measurement invariance (MI) was tested in CFA with ML estimator and FIML for estimating missing values. MI was investigated for three comparisons: between (1) GETs and SETs, (2) primary and secondary school teachers, and (3) German- and English-speaking teachers. For each comparison, four models with progressively stricter constraints were estimated (Putnick and Bornstein, 2016): (1) a configural model with no constraints, assuming only the factor structure is invariant; (2) a metric model with equal factor loadings; (3) a scalar model with equal factor loadings and intercepts; and (4) a residual model with equal factor loadings, intercepts, and residuals. The fit of the configural model was assessed using the above stated criterion for CFI, TLI, RMSEA, and SRMR. The metric, scalar, and residual models were each compared to the less restricted model using *χ*^2^-difference tests, as well as changes in CFI and RMSEA, with change cut-offs of ≥ −0.010 and ≥0.015, respectively (Chen, 2007). In cases where model comparison indicated that MI could not be assumed, we applied a backward-approach (Yoon and Millsap, 2007) to investigate whether partial metric or partial scalar invariance could be established, i.e., whether at least two factor loadings (partial metric) or two factor loadings and intercepts (partial scalar) per construct were invariant (Cieciuch and Davidov, 2015). In the empirical literature, it is argued that at least partial scalar invariance is necessary to allow for valid group comparisons (e.g., Byrne et al., 1989). However, it has also been questioned whether MI must be established at all for such comparisons (Robitzsch and Lüdtke, 2023). Data from the first two studies (Study 1a for questionnaire 1 and Study 1b for questionnaire 2), collected in German secondary schools, were used in the analyses of MI between GETs and SETs. This data was combined with data from Study 2, which focused on German primary school teachers, to asses MI between primary and secondary school teachers, and with data from Study 3 (an American sample), which was conducted with the translated questionnaire,^2^ to assess MI between German- and English-speaking teachers.

All analyses were conducted in R (R Core Team, 2022). For EFA, we used the package psych (Revelle, 2023; version 2.3.3) and for CFA, the package lavaan (Rossel, 2012; version 0.6–12).

## 3 Results

### 3.1 First short form questionnaire

#### 3.1.1 Item-analysis

The item analysis conducted in the first step based on the data from Study 1a (see Table 2) showed that no item had to be excluded. All items had an appropriate level of difficulty (>0.20 and <0.80). Moreover, the skewness and kurtosis of all items were < |2|. A maximum of 3% of the values were missing.

**Table 2:** Items on co-constructive collaboration and descriptive statistics (Questionnaire 1).

Items		*n*	*M*	*SD*	Skew	Kurtosis	Item difficulty
ccc01	The chemistry between us colleagues is good.	1,008	3.45	0.60	−0.69	0.09	0.69
ccc02	We have a trusting work environment.	1,007	3.46	0.62	−0.84	0.31	0.69
ccc03	We are equally involved in the collaboration.	1,002	2.95	0.84	−0.29	−0.77	0.59
ccc04	I can also act autonomously in the team.	1,003	3.44	0.63	−0.82	0.32	0.69
ccc05	During our team meetings, we work very intensively with one another.	987	3.08	0.78	−0.57	−0.09	0.62
ccc06	When we sit down together as a team, we negotiate shared goals.	991	3.21	0.74	−0.74	0.36	0.64
ccc07	We can stand disagreements and ambiguities in our team meetings.	978	3.15	0.70	−0.40	−0.33	0.63
ccc08	We develop and discuss shared plans of action.	990	3.24	0.71	−0.67	0.23	0.65
ccc09	Through our collaboration, we now take over many tasks that did not originally belong to our duties.	984	3.13	0.86	−0.59	−0.59	0.63
ccc10	Our collaboration has led us to ideas, that we would have never come up with alone.	985	2.98	0.79	−0.30	−0.57	0.60
ccc11	In the team, we know exactly what the other team members are up to.	986	2.72	0.79	−0.18	−0.40	0.54
ccc12	Working together does us good.	980	3.28	0.70	−0.69	0.25	0.66
ccc13	We all feel equally responsible for all students.	996	3.02	0.87	−0.47	−0.64	0.60
ccc14	As teachers, we feel we are equal.	996	3.30	0.81	−0.97	0.23	0.66

The items are the translated version from Study 3. The statistics are derived from Study 1a. The crossed-out items were excluded in the course of the analyses.

#### 3.1.2 Factor analyses

In the second step, the data from Study 1a was randomly halved into a training and test data set. Prior analyses proved the training data set to be suitable for EFA with KMO = 0.94 and a significant Bartlett’s Test of Sphericity (*p* < 0.001). A parallel factor analysis (Horn, 1965) was conducted on the 14 items on CCC (see Table 2), resulting in a model with four factors. Factor loadings of three items were <0.50 on any factor and were therefore excluded (ccc03, ccc09, ccc14). A subsequent parallel analysis with the remaining 11 items revealed three factors. Again, two items were excluded because of factor loadings <0.50 (ccc04, ccc07). The final exploratory model contained three factors with a total of nine items (see Supplementary Figure S1) which were interpreted as requirements for CCC (ccc01, ccc02), co-constructive activities (ccc05, ccc06, ccc08), and outcomes of CCC (ccc10, ccc11, ccc12c ccc13). The factor intercorrelations were *r* = 0.64 between requirements and activities, *r* = 0.71 between requirements and outcomes, and *r* = 0.86 between activities and outcomes.

**Figure 2:**
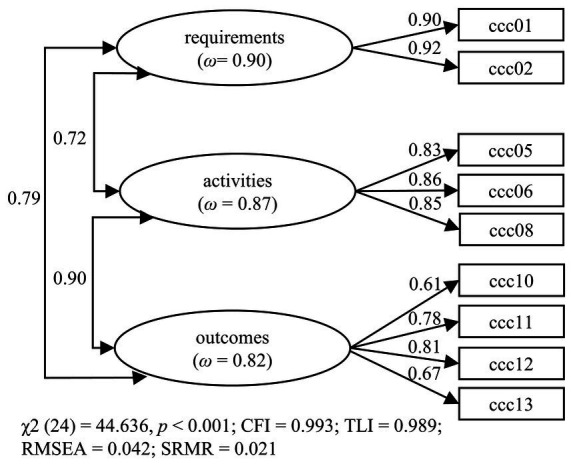
Results of the confirmatory factor analysis of the final three-factor model (Questionnaire 1).

The three-factor model with nine items was replicated with the independent test data set in a CFA. We specified a model with three correlated latent variables on which the corresponding items loaded (see Figure 2). The model demonstrated excellent fit, meeting the cut-off criteria for CFI, TLI, RMSEA, and SRMR, with all factor loadings > 0.50. However, the latent factor correlations were relatively high (0.72 ≤ *r* ≤ 0.90). An alternative model, which combined the two most highly correlated factors (activities and outcomes), provided an acceptable (*χ*^2^(26) = 103.684, *p* < 0.001, CFI = 0.972, TLI = 0.961, RMSEA = 0.077, SRMR = 0.032, factor intercorrelation = 0.77), but relatively weaker fit (∆*χ*^2^ = 50.792, ∆*df* = 2, *p* < 0.001). Therefore, the original three-factor model, as presented in Figure 2, was retained.

The final CFA model was replicated with data from Studies 1c, 1d, 2, and 3. All models showed a good fit (see Table 3). Only in Study 2 was RMSEA slightly above the cut-off of 0.06. The factor loadings, latent factor correlations, and omega were similar to those in the model above (see Figure 2) and can be found in Supplementary Figures S2–S5. It should be emphasized that the results thus also confirm the factor structure for German primary school teachers (2) and English-speaking teachers (Study 3).

**Table 3:** Results of the confirmatory factor analyses conducted with Questionnaire 1.

Study	*χ*^2^ (df)	*p* (*χ*^2^)	CFI	TLI	RMSEA	SRMR
1c	33.043 (24)	0.103	0.994	0.991	0.036 [0.000; 0.063]	0.023
1d	27.739 (24)	0.271	0.996	0.994	0.028 [0.000; 0.067]	0.026
2	72.376 (24)	<0.001	0.975	0.962	0.070 [0.052; 0.089]	0.028
3	48.790 (24)	0.002	0.988	0.982	0.046 [0.026; 0.065]	0.024

#### 3.1.3 Construct validity

In the third step, convergent construct validity was evaluated by examining correlations with related constructs. Table 4 presents the correlation coefficients between the three CCC scales and the validity constructs.

**Table 4:** Correlations of co-constructive collaboration and external variables (Questionnaire 1).

External variables	Co-constructive collaboration					
	Requirements		Actions		Outcomes	
	Study 1a	Study 3	Study 1a	Study 3	Study 1a	Study 3
Attitudes	0.09**	0.21***	0.11***	0.16***	0.16***	0.26***
Self-efficacy^1^	0.14***	0.27***	0.22***	0.27***	0.23***	0.32***
Responsibility—student achievement	0.08*	0.18***	0.18***	0.24***	0.18***	0.26***
Responsibility—student-teacher relationship	0.18***	0.17***	0.19***	0.22***	0.17***	0.22***
Co-teaching^2^						
One teach, one observe	−0.06	−0.07	−0.11 *	−0.08	−0.07	−0.04
One teach, one assist	0.04	−0.05	−0.0	0.05	−0.03	−0.01
Alternative teaching	0.21***	0.05	0.20***	0.10	0.14**	0.11
Parallel teaching	0.15**	0.03	0.16**	0.10	0.17**	0.13*
Station teaching	0.23***	−0.01	0.27***	0.05	0.26***	0.13*
Team teaching	0.15**	−0.05	0.23***	0.03	0.30***	0.09

*p < 0.05, **p < 0.01, ***p < 0.001. Spearman’s correlation coefficient was calculated for the co-teaching forms, while Pearson’s correlation coefficient was calculated for the remaining variables. ^1^Self-efficacy was measured differently in Study1a (self-efficacy in relation to inclusive teaching) and Study 3 (self-efficacy in relation to collaboration). ^2^In Study 1a, between 396 (for ‘parallel teaching’) and 420 (for ‘one teach, one assist’) of the 1,010 teachers provided information on co-teaching. In Study 3, 262 of the 481 teachers provided information on co-teaching.

The results demonstrate that for both, the German- (Study 1a) and the English-speaking sample (Study 3), each subscale of CCC correlated significantly with attitudes, self-efficacy, and teacher’s responsibility for student achievement and for their relationship with students. These correlations ranged from small to medium (0.08 ≤ *r* ≤ 0.32). For the co-teaching forms, the correlation between the German- and English-speaking samples differed. In the German-speaking sample (Study 1a), the three CCC scales were significantly related to the co-teaching forms ‘alternative teaching’, ‘parallel teaching’, ‘station teaching’, as well as ‘team teaching’. However, the co-teaching form ‘one teach, one assist’ did not correlate with any of the CCC scales and the form ‘one teach, one observe’ exhibited only a small correlation with co-constructive activities. In contrast, in the English-speaking sample (Study 3), there were only two significant correlations for outcomes of CCC with ‘parallel teaching’ and ‘station’ teaching.

#### 3.1.4 Measurement invariance

In the fourth step, we tested the final model for MI between GETs and SETs (data from Study 1a only), between primary and secondary school teachers (data from Studies 1a and 2), and between German- and English-speaking teachers (data from Studies 1a and 3). The results for MI between GETs and SETs (see Table 5, upper part) show that constraining the factor loadings to be equal (metric model) did not significantly worsen the model fit (*p* = 0.721) and improved both CFI and RMSEA. Therefore, metric invariance can be assumed. Further constraining the intercepts to be equal (scalar model) worsened the model fit significantly (*p* = 0.005). Although CFI and RMSEA worsened, the changes remained below the cut-offs. Given the significant difference in Chi^2^, we further tested for partial scalar invariance. We found that a model in which the constraints on the intercept parameters for items ccc10 and ccc13 were released did not fit significantly worse than the metric model (*p* = 0.392) and improved RMSEA, while CFI did not change. Therefore, partial scalar invariance can be assumed. Imposing constraints on the residuals within this partially restricted model (residual model) did not significantly worsen the model fit (*p* = 0.156). Although CFI and RMSEA worsened, the changes again remained below the cut-offs. Therefore, residual invariance can be assumed. It can be concluded that the factor loadings, and seven of the nine intercepts and residuals are measurement invariant between GETs and SETs.

**Table 5:** Results of the models testing for measurement invariance (Questionnaire 1).

Model	*χ*^2^ (df)	*p* (*χ*^2^)	CFI	TLI	RMSEA	SRMR	∆*χ*^2^ (∆df)	*p*(∆)	∆ CFI	∆ RMSEA
a) GETs vs. SETs										
Configural	83.694 (48)	0.001	0.993	0.990	0.038	0.019	–	–	–	–
Metric	88.103 (54)	0.002	0.994	0.992	0.035	0.021	3.673 (6)	0.721	0.001	−0.003
Scalar	106.459 (60)	<0.001	0.991	0.990	0.039	0.024	18.796 (6)	0.005	−0.003	0.004
Scalar partial^1^	92.093 (58)	0.003	0.994	0.992	0.034	0.022	4.104 (4)	0.392	0.000	−0.001
Residual	107.221 (65)	0.001	0.992	0.991	0.036	0.024	10.625 (7)	0.156	−0.002	0.002
b) Primary vs. secondary school teachers										
Configural	135.152 (48)	<0.001	0.988	0.982	0.051	0.021	–	–	–	–
Metric	147.057 (54)	<0.001	0.987	0.983	0.049	0.028	11.286 (6)	0.080	−0.001	−0.002
Scalar	190.845 (60)	<0.001	0.982	0.979	0.055	0.031	45.310 (6)	<0.001	−0.005	0.006
c) German- vs. English-speaking teachers										
Configural	111.566 (48)	<0.001	0.992	0.987	0.042	0.020	–	–	–	–
Metric	115.321 (54)	<0.001	0.992	0.989	0.039	0.022	3.556 (6)	0.737	0.000	−0.003
Scalar	161.655 (60)	<0.001	0.986	0.984	0.048	0.028	46.010 (6)	<0.001	−0.006	0.009
Scalar partial^1^	121.268 (57)	<0.001	0.991	0.989	0.039	0.023	5.927 (3)	0.115	−0.001	0.000
Residual	178.693 (63)	<0.001	0.985	0.982	0.050	0.024	42.122 (6)	<0.001	−0.006	0.011

1 The partially restricted scalar models were compared to the metric models.

The results for MI between primary and secondary school teachers (see Table 5, middle part) show that the metric model did not fit significantly worse than the configural model (*p* = 0.080). While RMSEA improved, CFI decreased slightly. However, the change remained below the cut-off. Therefore, metric invariance can be assumed. Further constraining the intercepts to be equal (scalar model) significantly worsened the model fit (*p* < 0.001). Although CFI and RMSEA worsened as well, the changes remained below the cut-offs. Given the significant difference in Chi^2^, we further tested for partial scalar invariance. However, we were unable to identify a model that met the requirements for partial scalar invariance. Hence, only the factor loadings are measurement invariant between primary and secondary school teachers.

The results for MI between German- and English-speaking teachers (see Table 5, lower part) show that constraining the factor loadings to be equal (metric model) did not significantly worsen the model fit (*p* = 0.737). While RMSEA improved, CFI did not change. Therefore, metric invariance can be assumed. Constraining the intercepts to be equal (scalar model) worsened the model fit significantly (*p* < 0.001). Although CFI and RMSEA worsened as well, the changes remained below the cut-offs. Given the significant difference in Chi^2^, we further tested for partial scalar invariance and found that a model in which the constraints on the intercept parameters for items ccc08, ccc10, and ccc11 were released, did not fit significantly worse than the metric model (*p* = 0.115). While RMSEA remained unchanged, CFI decreased slightly. However, the change was below the cut-off. Therefore, partial scalar invariance can be assumed. Further imposing constraints on the residuals within this partially restricted scalar model (residual model) led to a significantly worse model fit (*p* < 0.001). Both CFI and RMSEA worsened, with the changes falling below the cut offs. Because of the significant difference in Chi^2^, residual invariance cannot be assumed. It can be concluded that the factor loadings, and six of the nine intercepts are measurement invariant between German- and English-speaking teachers.

### 3.2 Second short form questionnaire

#### 3.2.1 Item analysis

The item analysis conducted in step one based on the data from Study 1b (see Table 6) showed that no item had to be excluded. All items had an appropriate level of difficulty (>0.20 and <0.80). Moreover, the skewness and kurtosis of all items were <|2|. Between 2 and 8% of the values were missing.

**Table 6:** Items on co-constructive collaboration and descriptive statistics (Questionnaire 2).

Items		*n*	*M*	*SD*	Skew	Kurtosis	Item difficulty
ccc15	In the team there is a balanced giving and taking.	491	3.15	0.73	−0.52	−0.09	0.63
ccc16r	In our team, one person decides in which direction to go.	491	3.23	0.73	−0.63	−0.05	0.65
ccc17r	With my team, I can do things that I cannot do alone.	492	3.00	0.74	−0.56	0.31	0.60
ccc18r	I am not reliant on the skills available in the team.	490	3.06	0.78	−0.51	−0.21	0.61
ccc19r	In the team, we sometimes work on the same thing for a long time to optimise it.	491	2.53	0.79	−0.10	−0.43	0.51
ccc20	Concepts or methods that we are currently developing are revised numerous times in our team.	488	2.55	0.81	−0.30	−0.44	0.51
ccc21	When differences of opinion arise in our team, we try to understand the viewpoint of the other person.	489	3.23	0.65	−0.80	1.61	0.65
ccc22	In the team, we reflect regularly on the successes and failures of our work.	488	2.81	0.86	−0.40	−0.45	0.56
ccc23	We have developed in the team a shared understanding of our work.	487	3.06	0.75	−0.64	0.38	0.61
ccc24	All team members feel personally committed to the realisation of our ideas.	484	2.81	0.81	−0.33	−0.36	0.56
ccc25	Team members who previously had a different profession also take over tasks from other areas.	460	2.70	0.81	−0.40	−0.23	0.54
ccc26	I continuously adapt the concepts and methods developed in the team to everyday school life.	485	2.82	0.71	−0.61	0.61	0.56
ccc27	Through team collaboration, I have more time for other things.	487	2.48	0.89	0.03	−0.73	0.50
ccc28r	At home, I must also think about the difficulties of working as a team.	487	3.07	0.89	−0.55	−0.66	0.61

The items are the translated version from Study 3. The statistics are derived from Study 1b. Items ccc16r, ccc18r, and ccc28r are negatively formulated items that were recoded for the analyses in favour of a consistent polarity. The crossed-out items were excluded in the course of the analyses.

#### 3.2.2 Factor analyses

In step two, the data from Study 1b was randomly halved into a training and test data set. Prior analyses proved the training data set to be suitable for EFA with KMO = 0.87 and a significant Bartlett’s Test of Sphericity (*p* < 0.001). A parallel factor analysis was conducted on the 14 items on CCC (see Table 6) resulting in a model with two factors. However, six items with communalities <0.40 had to be excluded (ccc16, ccc18, ccc19, ccc25, cccc27, ccc28). A subsequent parallel analysis with the remaining eight items revealed again two factors. One item had to be excluded because of factor loadings <0.50 on both factors (ccc17). The final exploratory model contained two factors with a total of seven items (see Supplementary Figure S6) which were interpreted as team commitment (ccc15, ccc21, ccc23, ccc24) and iterative revisions (ccc20, ccc22, ccc26). The factor intercorrelation was *r* = 0.51.

**Figure 3:**
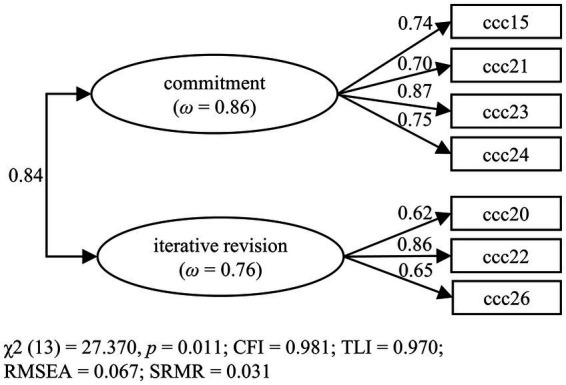
Results of the confirmatory factor analysis of the final two-factor model (Questionnaire 2).

The two-factor model with seven items was replicated with the test data set in a CFA. We specified a model with two correlated latent variables on which the corresponding items loaded (see Figure 3). The model showed a good fit, with all factor loadings > 0.50 and only RMSEA being slightly above the cut-off of 0.06. Latent factor correlation between commitment and revisions was 0.84. However, an alternative model containing only one factor (*χ*^2^ (14) = 59.475, *p* < 0.001, CFI = 0.941, TLI = 0.911, RMSEA = 0.115, SRMR = 0.047) resulted in a significantly worse model fit (∆*χ*^2^ = 20.092, ∆*df* = 1, *p* < 0.001). Therefore, the original two-factor model as shown in Figure 3 was retained.

The final CFA model was replicated with data from Studies 1c, 1d, 2, and 3. All models show a good fit (see Table 7). Only in Studies 1c and 1d is RMSEA again above the cut-off of 0.06. The factor loadings, latent factor correlations, and omega are similar to the ones in the model above (see Figure 3) and can be found in Supplementary Figures S7–S10. It should be emphasized that the results thus also confirm the factor structure for German primary school teachers (Study 2) and for English-speaking teachers (Study 3).

**Table 7:** Results of the confirmatory factor analyses conducted with Questionnaire 2.

Study	*χ*^2^ (df)	*p* (*χ*^2^)	CFI	TLI	RMSEA	SRMR
1c	44.500 (13)	<0.001	0.966	0.945	0.091 [0.063; 0.122]	0.030
1d	29.683 (13)	0.005	0.973	0.957	0.082 [0.043; 0.121]	0.031
2	19.524 (13)	0.108	0.994	0.991	0.036 [0.000; 0.066]	0.020
3	25.805 (13)	0.018	0.991	0.985	0.045 [0.018; 0.071]	0.019

#### 3.2.3 Construct validity

In the third step, convergent construct validity was evaluated by examining correlations with related constructs. Table 8 presents the correlation coefficients between the two CCC scales and the validity constructs.

**Table 8:** Correlations of co-constructive collaboration and external variables (Questionnaire 2).

External variables	Co-constructive collaboration			
	Commitment		Iterative revision	
	Study 1b	Study 3	Study 1b	Study 3
Attitudes	0.18***	0.21***	0.22***	0.24***
Self-efficacy^1^	0.21***	0.30***	0.23***	0.31***
Responsibility—student achievement	0.10*	0.20***	0.13**	0.28***
Responsibility—student-teacher relationship	0.15**	0.21***	0.16***	0.20***
Co-teaching^2^				
One teach, one observe	−0.06	−0.04	0.08	−0.08
One teach, one assist	0.08	−0.02	0.11	−0.04
Alternative teaching	0.21**	0.07	0.18*	0.17*
Parallel teaching	0.11	0.11	18**	0.04
Station teaching	0.31***	0.07	0.46***	0.05
Team teaching	0.20**	0.09	0.29***	0.09

*p < 0.05, **p < 0.01, ***p < 0.001. Spearman’s correlation coefficient was calculated for the co-teaching forms, while Pearson’s correlation coefficient was calculated for the remaining variables. ^1^Self-efficacy was measured differently in Study 1b (self-efficacy in relation to inclusive teaching) and Study 3 (self-efficacy in relation to collaboration). ^2^In Study 1b, between 187 (for ‘one teach, one observe’) and 192 (for ‘team teaching’) of the 493 teachers provided information on co-teaching. In Study 3, 262 of the 481 teachers provided information on co-teaching.

The results demonstrate that for both the German- (Study 1b) and the English-speaking sample (Study 3), both subscales of CCC correlated significantly with attitudes, self-efficacy, and teachers’ responsibility for student achievement and for their relationship with students. These correlations ranged from small to medium (0.10 ≤ *r* ≤ 0.31). For the co-teaching forms, the correlation between the German- and English-speaking sample differed again. In the German-speaking sample (Study 1b), the two CCC scales were significantly related to the co-teaching forms ‘alternative teaching’, ‘station teaching’, as well as ‘team teaching’. However, the co-teaching forms ‘one teach, one observe’ and ‘one teach, one assist’ did not correlate with the CCC scales and the co-teaching form ‘parallel teaching’ exhibited only a small correlation with iterative revision, but not commitment. In contrast, in the English-speaking sample (Study 3), there was only one significant correlation between iterative revision and ‘alternative teaching’.

#### 3.2.4 Measurement invariance

In step four, we tested the final model for MI between GETs and SETs (data from Study 1b only), between primary and secondary school teachers (data from Studies 1b and 2), and between German- and English-speaking teachers (data from Studies 1b and 3). The results for MI between GETs and SETs (see Table 9, upper part) show that full MI can be assumed, as each model did not fit significantly worse than the less restricted model (all *p* > 0.05), and RMSEA improved across all models. Moreover, CFI changed only minimally across the models, with changes being positive or remaining below the cut-off. Hence, it can be concluded that factor loadings, intercepts, and residuals are invariant between GETs and SETs.

**Table 9:** Results of the models testing for measurement invariance (Questionnaire 2).

Model	*χ*^2^ (df)	*p* (*χ*^2^)	CFI	TLI	RMSEA	SRMR	∆*χ*^2^ (∆df)	*p*(∆)	∆ CFI	∆ RMSEA
a) GETs vs. SETs										
Configural	69.654 (26)	<0.001	0.969	0.949	0.083	0.039	–	–	–	–
Metric	75.618 (31)	<0.001	0.968	0.956	0.076	0.044	4.619 (5)	0.464	−0.001	−0.007
Scalar	78.480 (36)	<0.001	0.969	0.964	0.069	0.045	2.818 (5)	0.728	0.001	−0.007
Residual	88.008 (43)	<0.001	0.968	0.968	0.065	0.048	8.331 (7)	0.304	−0.001	−0.004
b) Primary vs. secondary school teachers										
Configural	65.931 (26)	<0.001	0.984	0.974	0.059	0.028	–	–	–	–
Metric	96.603 (31)	<0.001	0.974	0.964	0.069	0.057	28.360 (5)	<0.001	−0.010	0.010
Partial metric^1^	66.334 (29)	<0.001	0.985	0.978	0.054	0.028	0.339 (3)	0.953	0.001	−0.005
Scalar	98.142 (32)	<0.001	0.973	0.965	0.068	0.037	31.813 (3)	<0.001	−0.012	−0.014
c) German- vs. English-speaking teachers										
Configural	72.213 (26)	<0.001	0.983	0.973	0.060	0.026	–	–	–	–
Metric	100.749 (31)	<0.001	0.975	0.966	0.068	0.052	25.100 (5)	<0.001	−0.008	0.008
Partial metric^1^	75.577 (29)	<0.001	0.983	0.976	0.057	0.031	2.953 (3)	0.399	0.008	−0.003
Scalar	153.858 (32)	<0.001	0.956	0.953	0.088	0.45	86.931 (3)	<0.001	−0.027	0.031

1 The partially restricted metric models were compared to the configural models.

The results for MI between primary and secondary school teachers (see Table 9, middle part) show that the metric model fitted significantly worse than the configural model (*p* < 0.001). CFI decreased by the cut-off of −0.010. RMSEA also worsened but remained below the cut-off. Due to the decrease in CFI and the significant difference in Chi^2^, we further tested for partial metric invariance and found that a model in which the constraints on the factor loadings of items ccc15 and ccc20 were released did not worsen the model fit compared to the configural model (*p* = 0.953). Moreover, CFI and RMSEA improved. Therefore, partial metric invariance can be assumed. Restricting the intercepts in this partial metric model (scalar model) resulted in a significantly worse model fit (*p* < 0.001) and worsened CFI as well as RMSEA. The changes were just below or above the cut-offs. Therefore, we further tested for partial scalar invariance, but were unable to identify a model that met the requirements for partial scalar invariance. Hence, only partial metric MI with two factor loadings functioning differently between primary and secondary school teachers can be assumed.

The results for MI between German- and English-speaking teachers (see Table 9, lower part) show that constraining the factor loadings to be equal (metric model) significantly worsened the model fit (*p* < 0.001). Although CFI and RMSEA worsened, the changes remained below the cut-offs. Given the significant difference in Chi^2^, we further tested for partial metric invariance and found that a model in which the constraints on the loadings of items ccc22 and ccc23 were released, did not worsen the model fit compared to the configural model (*p* = 0.399). Moreover, both CFI and RMSEA improved. Therefore, partial metric invariance can be assumed. Further restricting the intercepts in this partial metric model (scalar model) led to a significantly worse model fit (*p* < 0.001). In addition, both CFI and RMSEA worsened, with changes clearly exceeding the cut-offs. Consequently, we tested for partial scalar invariance, but were unable to identify a model that met the requirements for partial scalar invariance. Hence, only partial metric MI, with two factor loadings functioning differently between German- and English-speaking teachers, can be assumed.

## 4 Discussion

The aim of this study was to develop and evaluate psychometrically sound and theory-based questionnaires to properly understand and evaluate teacher collaboration in inclusive schools. Drawing on the theoretical framework by Grosche et al. (2020), two short questionnaires were created to capture co-constructive collaboration as a particularly intensive and, at the same time, especially promising form of collaboration with regard to the effective implementation of inclusive education. Both scales were tested in German and English to provide a basis for future cross-cultural studies on this form of collaboration.

In our analyses, we first investigated whether a reliable and substantial factor structure could be identified that adequately measures the main dimensions of the model (research question 1). For this purpose, we used a sample from German secondary school teachers and replicated the model with other samples, including English-speaking teachers, for whom the translated English version of the questionnaire was used. We then examined the construct validity for both the German and the English version of the questionnaire (research question 2) and tested, whether the measurement model was invariant between (1) GETs and SETs, (2) primary and secondary school teachers, and (3) German- and English-speaking teachers (research question 3).

The results for research question 1 yielded two short questionnaires measuring different dimensions of CCC between GETs and SETs in inclusive schools. The first questionnaire provides a more comprehensive view on CCC and covers three main dimensions of the theory: requirements, co-constructive activities, and outcomes. The second questionnaire focuses more specifically on teachers’ personal commitment to CCC and on iterative revision as a specific form of co-constructive activity.

Within the factor structures, we found that some of the subscales were highly correlated with each other. This result aligns well with theoretical expectations (Grosche et al., 2020). For example, the high correlation observed between “outcomes” and “requirements” can be theoretically justified by the cyclical nature of CCC. According to Grosche et al. (2020), the outcomes of collaboration can influence future requirements as well as activities. Thus, while the high intercorrelation among the subscales may raise questions about redundancy, it reflects the intertwined nature of these dimensions within the theory of CCC. Nonetheless, the results indicate that the dimensions should remain distinct, as neither a two-factor model (for questionnaire 1) nor a single-factor model (for questionnaire 2) provided a better fit.

Furthermore, as the aim was to develop short questionnaires, our developed measures do not reflect the theory in every detail. For example, we did not take school conditions into account and did not differentiate between specific and general requirements of the CCC theory. Moreover, within the identified dimensions, not all relevant aspects are covered. For example, the subscale ‘requirements’ (questionnaire 1) focuses on a trusting work atmosphere. Despite these limitations/reductions in theory, we find that the subscales identified—along with their respective items—capture the core characteristics of CCC effectively. Compared to other forms of collaboration, CCC necessitates high levels of commitment (subscale questionnaire 2) as well as trust (item of requirements, questionnaire 1) and is marked by intensive collaborative activities, such as iterative revisions (subscale questionnaire 2) or processes of negotiation and joint planning (items of co-constructive activities, questionnaire 1). These activities are correlated with outcomes such as the generation of new ideas or a shared responsibility for all students (items of outcomes, questionnaire 1). We therefore consider the questionnaires as suitable for capturing the core elements of the CCC theory. Should future studies require a more comprehensive assessment of CCC, the long form of the questionnaire could be used, though it has so far only been validated in German (Fussangel et al., 2023). The long form of the questionnaire consists of 52 items in total. General requirements, for example, are measured by 10 items that reflect the three sub-dimensions of school conditions, attitude towards collaboration, and experiences with collaboration.

The results concerning construct validity (research question 2) showed that the identified scales of CCC correlate with attitudes towards inclusion, teachers’ responsibilities, and their self-efficacy, thus confirming the existence of construct validity. This applies to both the German- and the English-speaking sample. With regard to the co-teaching forms, however, the results were divergent. For the German-speaking sample, there is a tendency that CCC does not correlate with the two co-teaching forms in which only one teacher teaches and the other observes or assists. However, in the forms in which both teachers play an active role in teaching, positive correlations were found. This confirms construct validity, as these forms require more negotiation processes as well as joint planning. For the English-speaking sample, however, there were only isolated correlations.

The results for MI (Research Question 3) indicate that both questionnaires allow valid group comparisons between GETs and SETs. Questionnaire 1 achieved partial scalar invariance, while questionnaire 2 demonstrated full scalar invariance between GETs and SETs. These results support the content validity of the questionnaires, which were specifically designed to assess the collaboration between these two groups of teachers. Consequently, the questionnaires can be practically applied to identify and analyse differences in GETs’ and SETs’ perceptions of collaboration.

In contrast to these findings, the results for MI between primary and secondary school teachers as well as German- and English-speaking teachers were less satisfactory. While partial scalar invariance could be established in the first questionnaire for German- and English-speaking teachers, there were considerable differences in the intercepts for these groups in the second questionnaire, as well as between primary and secondary school teachers in both questionnaires. In these latter cases, the requirements for partial scale invariance were not met and only (partial) metric invariance could be established. Although the extent to which these differences truly impact the validity of group comparisons (e.g., Robitzsch and Lüdtke, 2023) cannot be fully determined at this point, the variations in factor loadings or intercepts suggest that certain items may be interpreted differently across different contexts. Nevertheless, it is encouraging that the factor structure for collaboration between primary school teachers and English-speaking teachers was satisfactory, indicating that CCC can still be measured reliably in these contexts.

Based on the results, we consider the questionnaires as suitable for capturing CCC of GETs and SETs. The questionnaires can be applied in schools and professional teacher development, serving as tools for diagnosis, reflection, and evaluation. For example, they can help identify areas where collaboration is less well developed or differently perceived by GETs and SETs, inform the design of targeted training initiatives, and subsequently be used to evaluate their effectiveness. Due to their brevity, the instruments are particularly well suited for such evaluative purposes and for longitudinal analyses in general, which also allow for an examination of the causal assumptions of the CCC theory and, thus, for continued theoretical development.

With regard to the aim of supporting cross-cultural studies, it can be summarised that the questionnaires could be validated both in German and English, although a direct comparison is not always possible due to limited MI As a limitation it should also be noted that the English-language sample consisted solely of teachers in the USA. As such, our analyses represent an *initial* step towards cross-cultural validation. Nonetheless, the United States provides a useful starting point due to the comparability of key aspects of inclusive education (such as the role of SETs or the importance of collaboration, e.g., Björn et al., 2016; Neumann and Lütje-Klose, 2021) with other countries and educational systems. More fundamentally, the use of English ensures that the questionnaire is easily accessible for scientific purposes, facilitating wider dissemination and enabling comparisons across international research studies. To further establish the external validity of our developed measures, future studies could replicate our findings in other English-speaking countries (e.g., Canada, England, and Australia) as well as in other languages and countries.

In addition to the aforementioned limitations associated with the development of a short questionnaire and the English-language sample, further limitations must be considered. First, although longitudinal data were available, this paper focused on MI between groups. Nevertheless, for a more comprehensive longitudinal examination of collaboration, it would be desirable to assess MI over time (cf. Mackinnon et al., 2022).

Second, although we differentiated between primary and secondary schools in the German-speaking sample, we did not apply this distinction to the English-speaking sample, because the sample size would have been too small. Additionally, we did not differentiate between lower and advanced secondary schools within the German- or English-speaking samples. Previous studies indicate that differences in collaboration can be observed across lower and advanced secondary schools (Mora-Ruano et al., 2018; Pozas and Letzel-Alt, 2023; Webs and Holtappels, 2018). Thus, future work could build on our work by testing the efficacy of our questionnaires across these school contexts.

Third, it is noteworthy that the item means consistently exceeded the scale midpoint, indicating that teachers collaborate in a distinctly co-constructive manner. This contradicts previous findings, which suggests that CCC is less frequently implemented in schools (e.g., Pozas and Letzel-Alt, 2023; Webs and Holtappels, 2018). A potential explanation for this discrepancy may be the influence of social desirability. Additionally, because only CCC was assessed in the current study, teachers may have overestimated their level of collaboration without contrasting it against less intensive forms of collaboration (Kluge et al., in print). Future research should address these considerations by exploring potential response biases and including comparative measures of various collaboration forms.

Despite these limitations, the results demonstrate the reliability and validity of the instruments developed for measuring CCC between GETs and SETs in inclusive schools. The instruments provided allow for an economical yet meaningful assessment of the core aspects of CCC. With the validation of a German and an English version of the questionnaire, our study invites cross-cultural research into CCC in different national and demographic contexts.

## Data Availability

The datasets presented in this article are not readily available. The public user file for the INSIDE datasets will be available in 2025 (https://doi.org/10.5157/INSIDE:1.0.0). The other datasets presented in this article can be provided upon request. Requests to access these datasets should be directed to jkluge@uni-wuppertal.de.

## Footnotes

^1^Data collection involved a two-step process. In the first step, an initial pilot study (*N* = 902) was conducted to find teachers working in inclusive schools in which collaboration between teachers occurs. In step two, participants of the pilot study who fit these requirements (*n* = 644) were invited to complete Study 3 one week later. Of the 644 individuals invited, 542 (84.16%) returned to complete Study 3. Of these, *n* = 481 worked as either a GET or SET.

^2^The German versions of the two questionnaires were translated into English using forward-backward translation (Brislin, 1980), by a native German speaker fluent in English and a native English speaker fluent in German.
