# Supplementary figures and images for: Measuring co-constructive collaboration between general and special education teachers in inclusive schools—development and validation of two short questionnaires
Source: Front Psychol. 2025 Jun 17;16:1535727. doi: 10.3389/fpsyg.2025.1535727 (PMC12209277; doi:10.3389/fpsyg.2025.1535727)

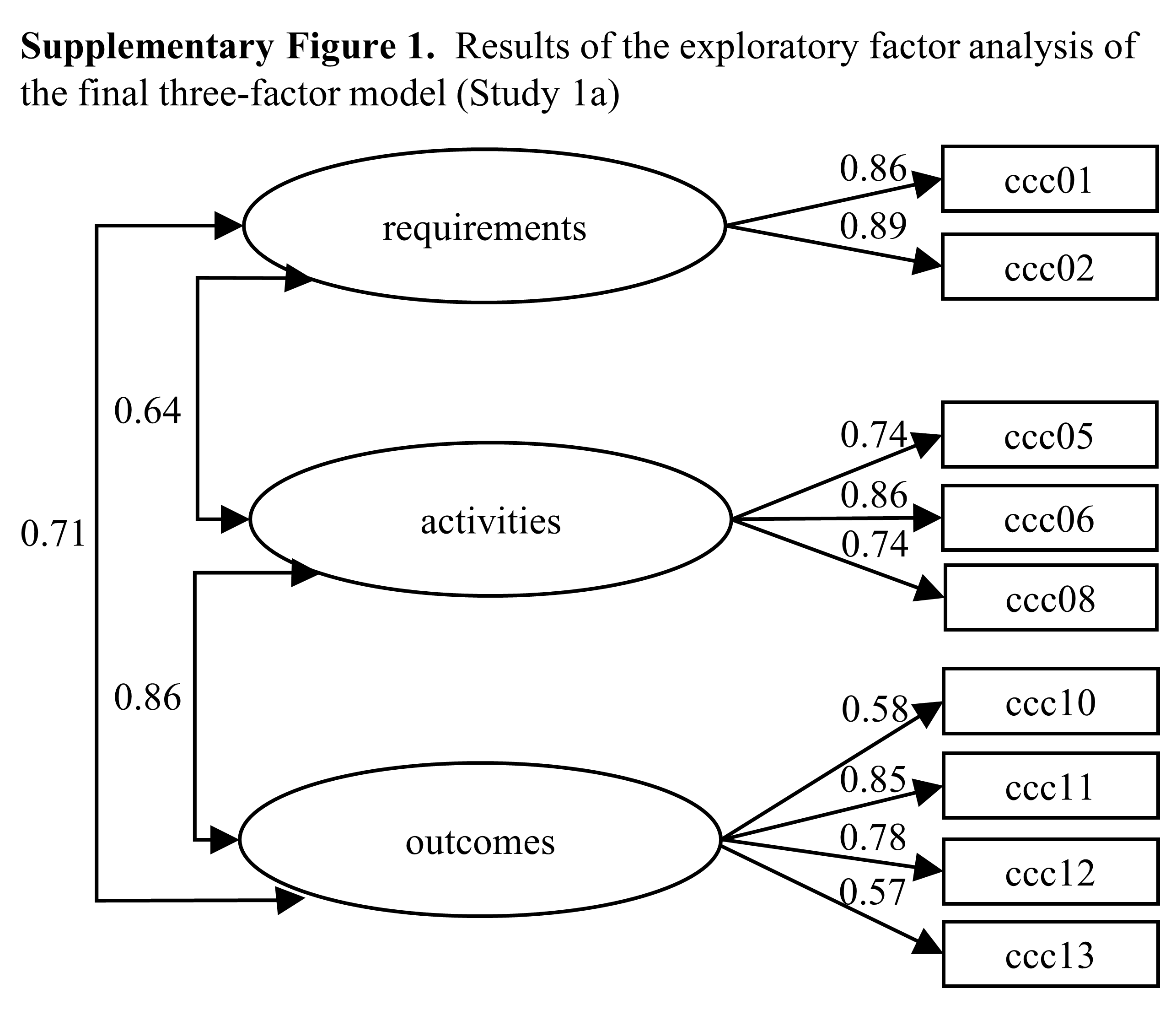

Supplement: Supplementary file 1 [file Image_1.tif]

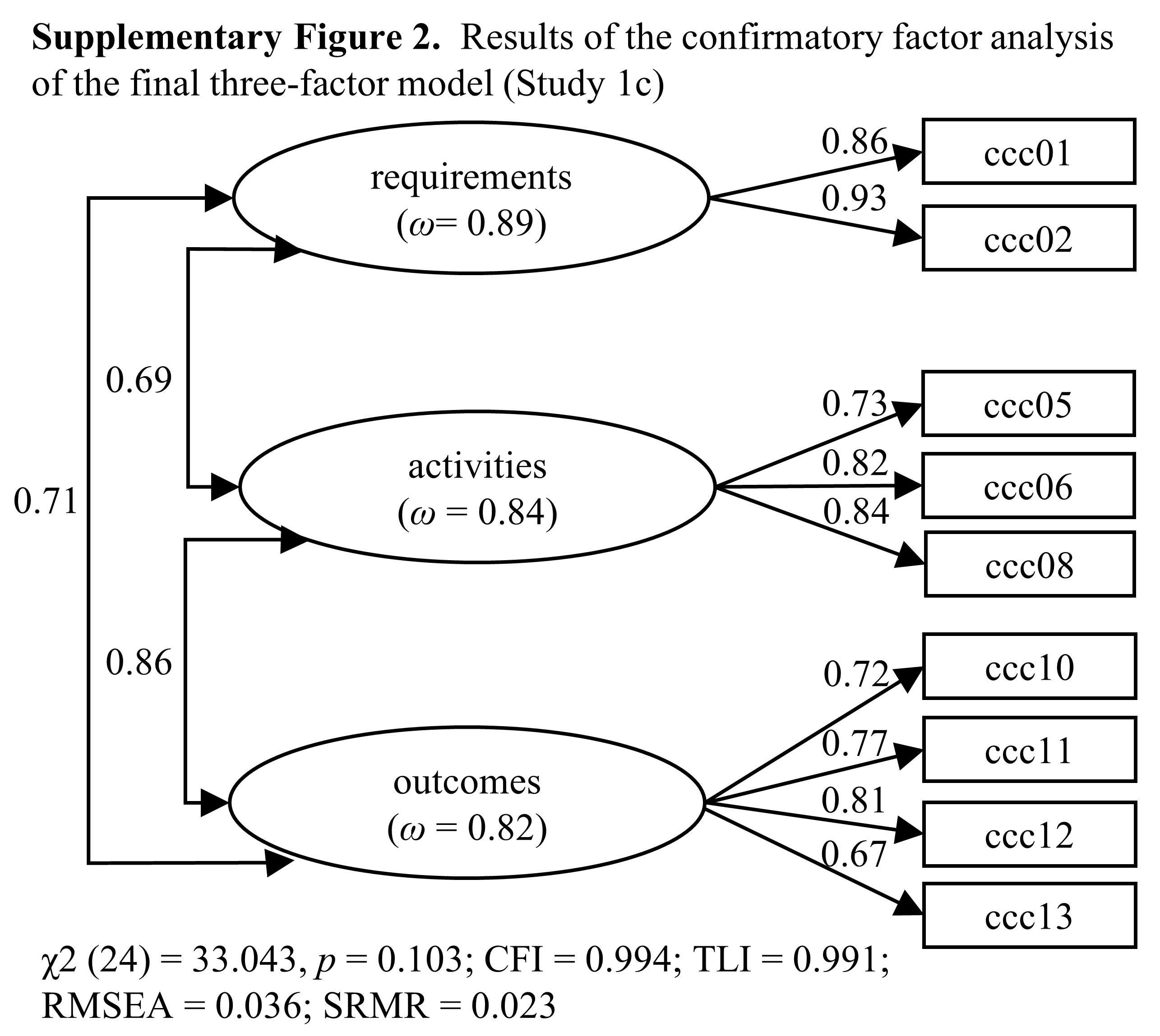

Supplement: Supplementary file 2 [file Image_2.tif]

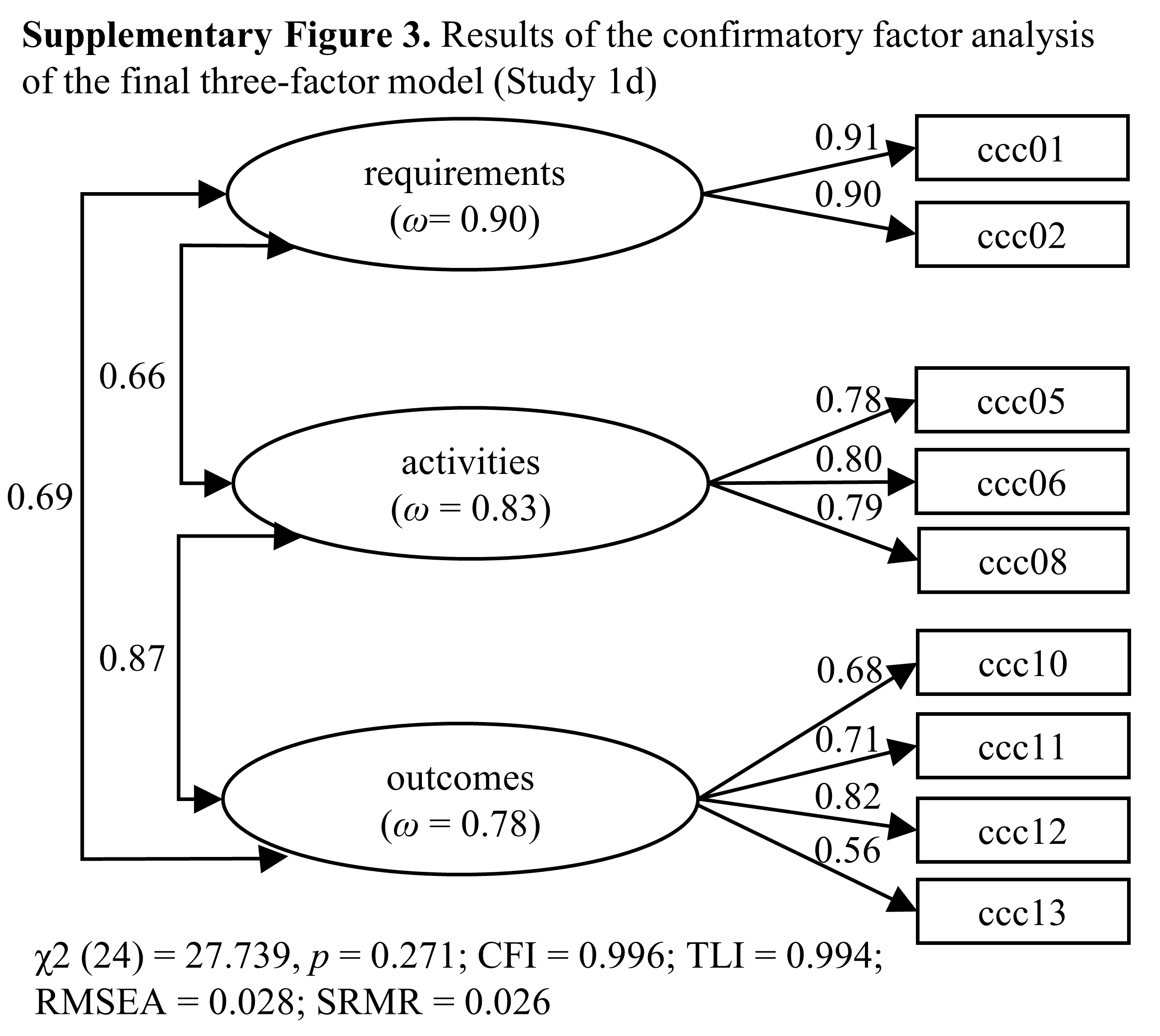

Supplement: Supplementary file 3 [file Image_3.tif]

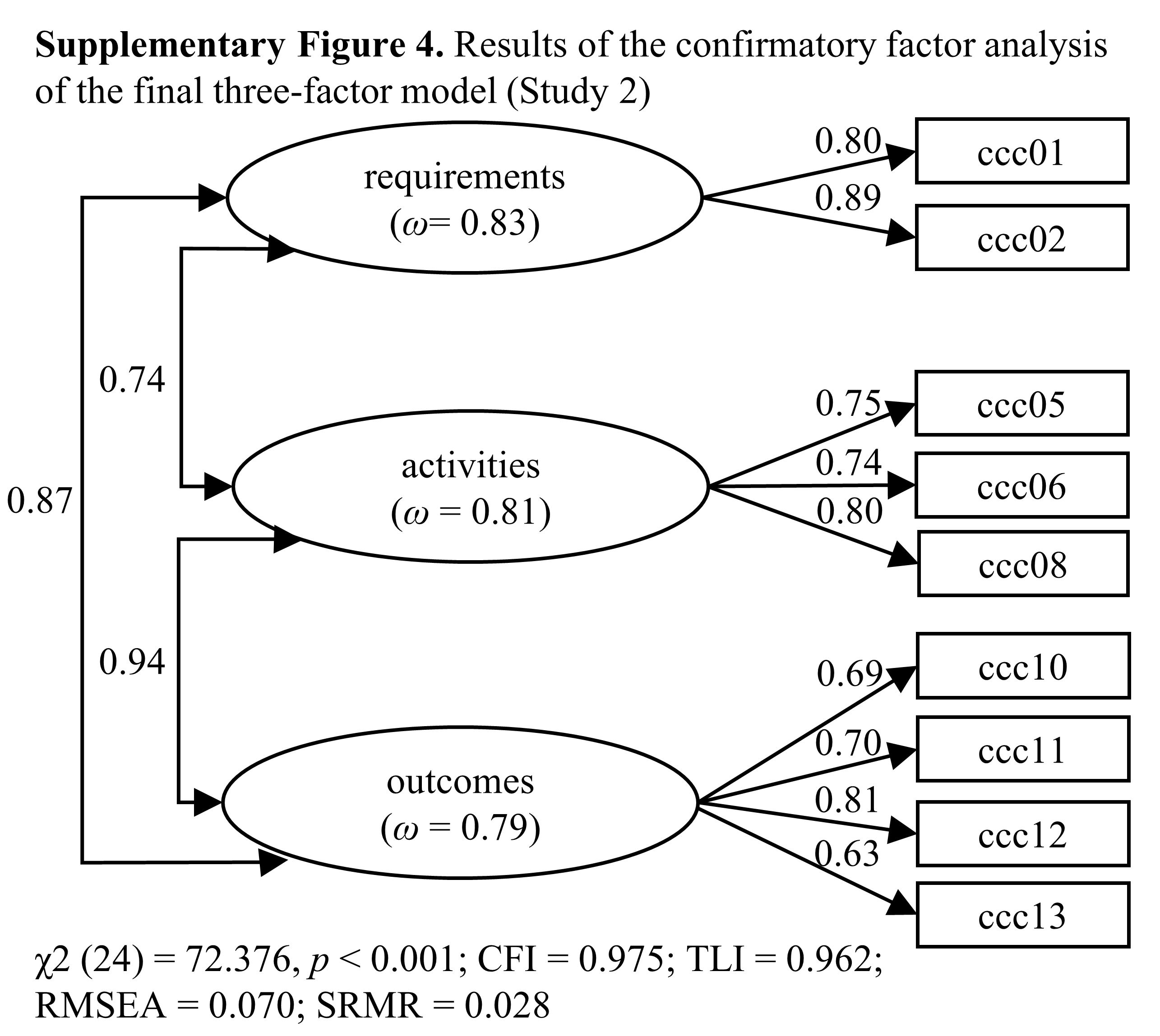

Supplement: Supplementary file 4 [file Image_4.tif]

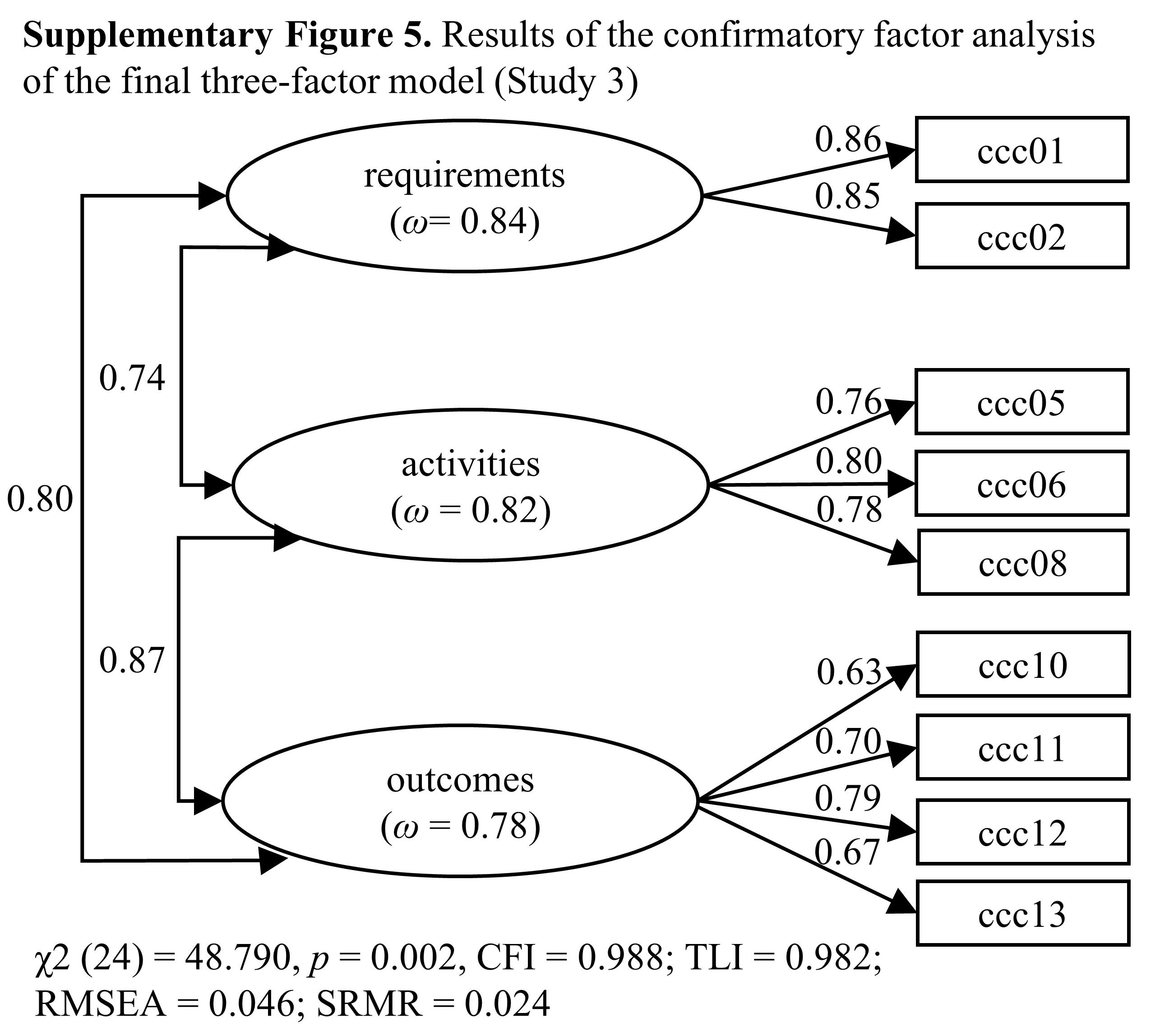

Supplement: Supplementary file 5 [file Image_5.tif]

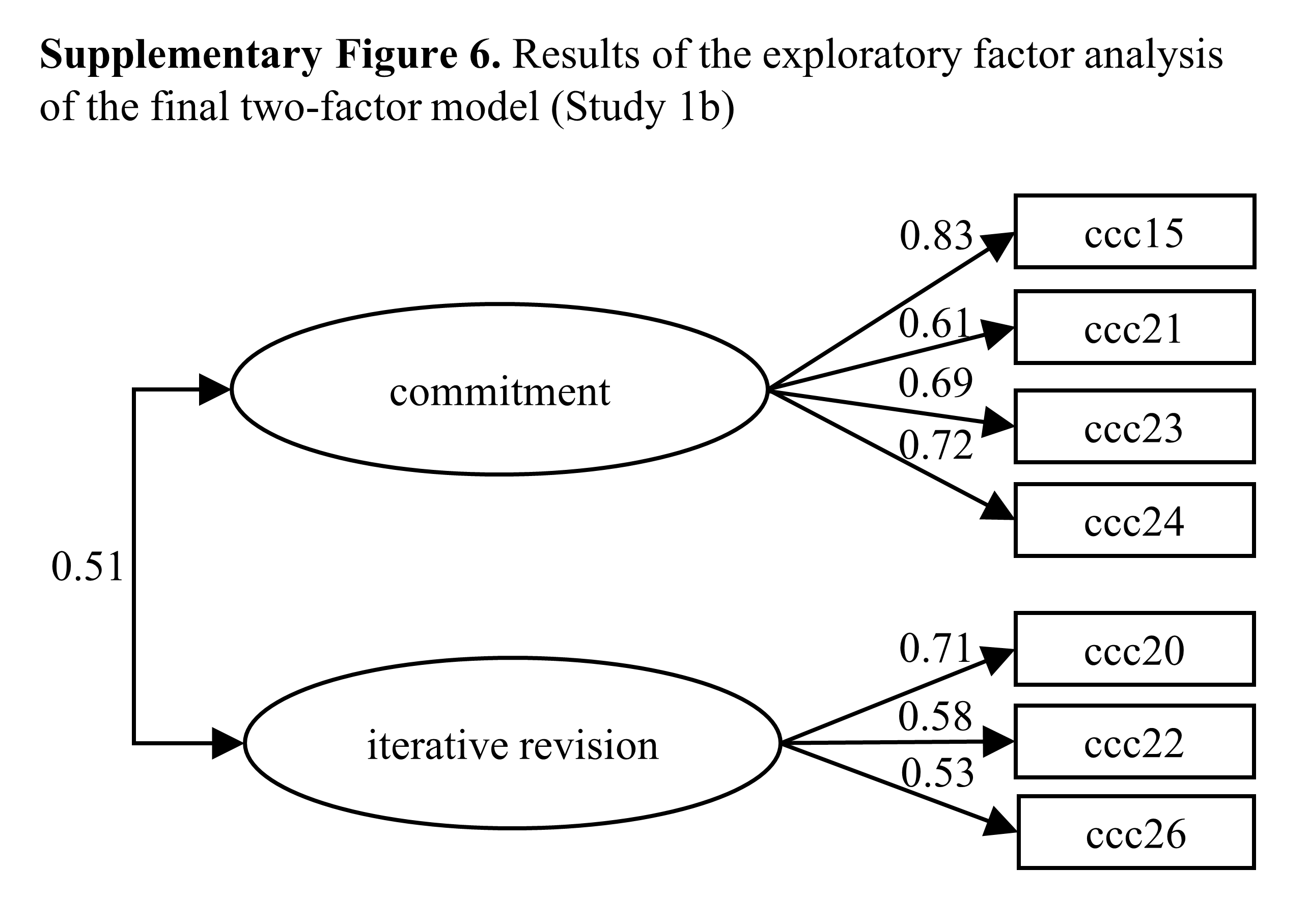

Supplement: Supplementary file 6 [file Image_6.tif]

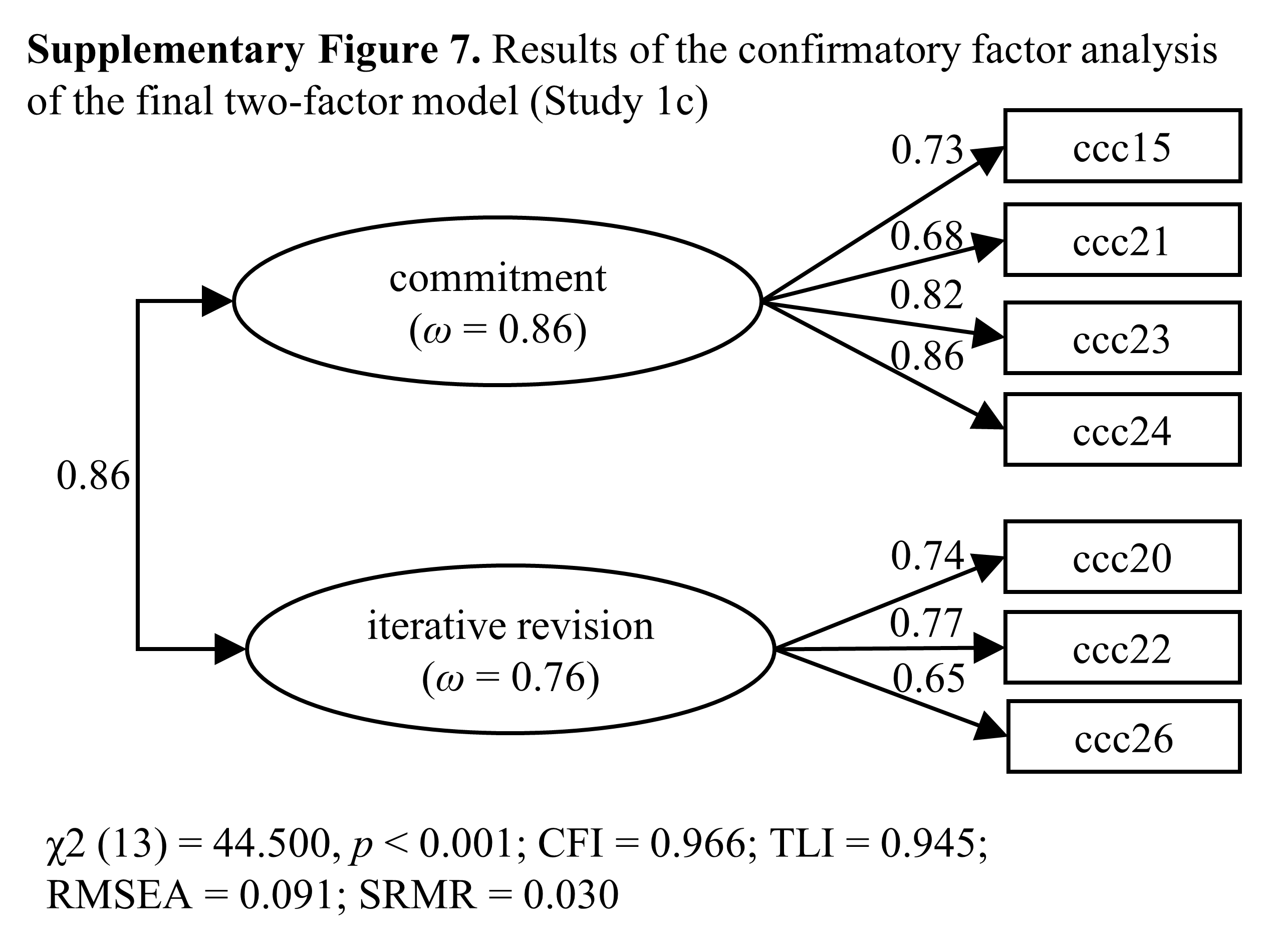

Supplement: Supplementary file 7 [file Image_7.tif]

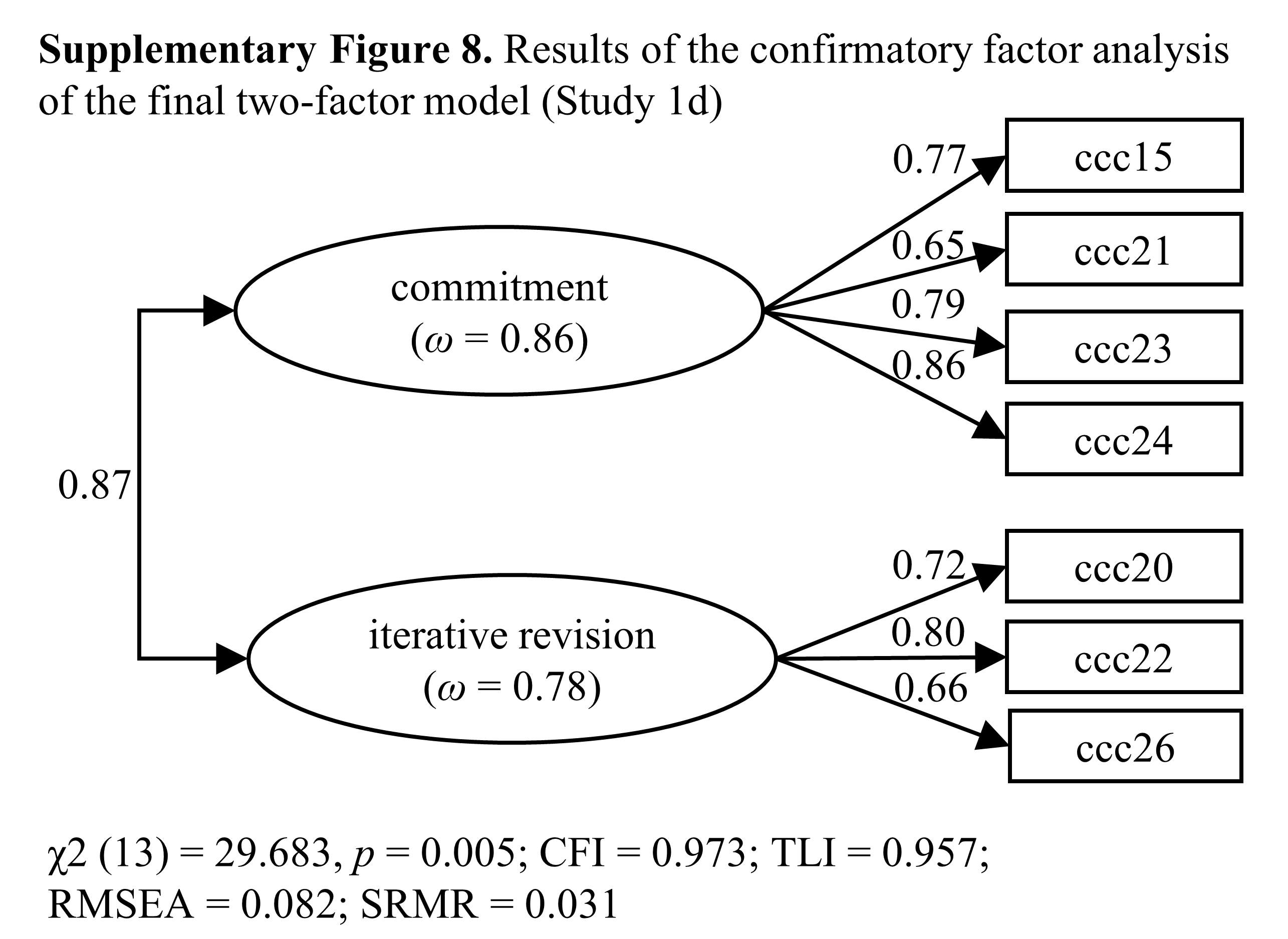

Supplement: Supplementary file 8 [file Image_8.tif]

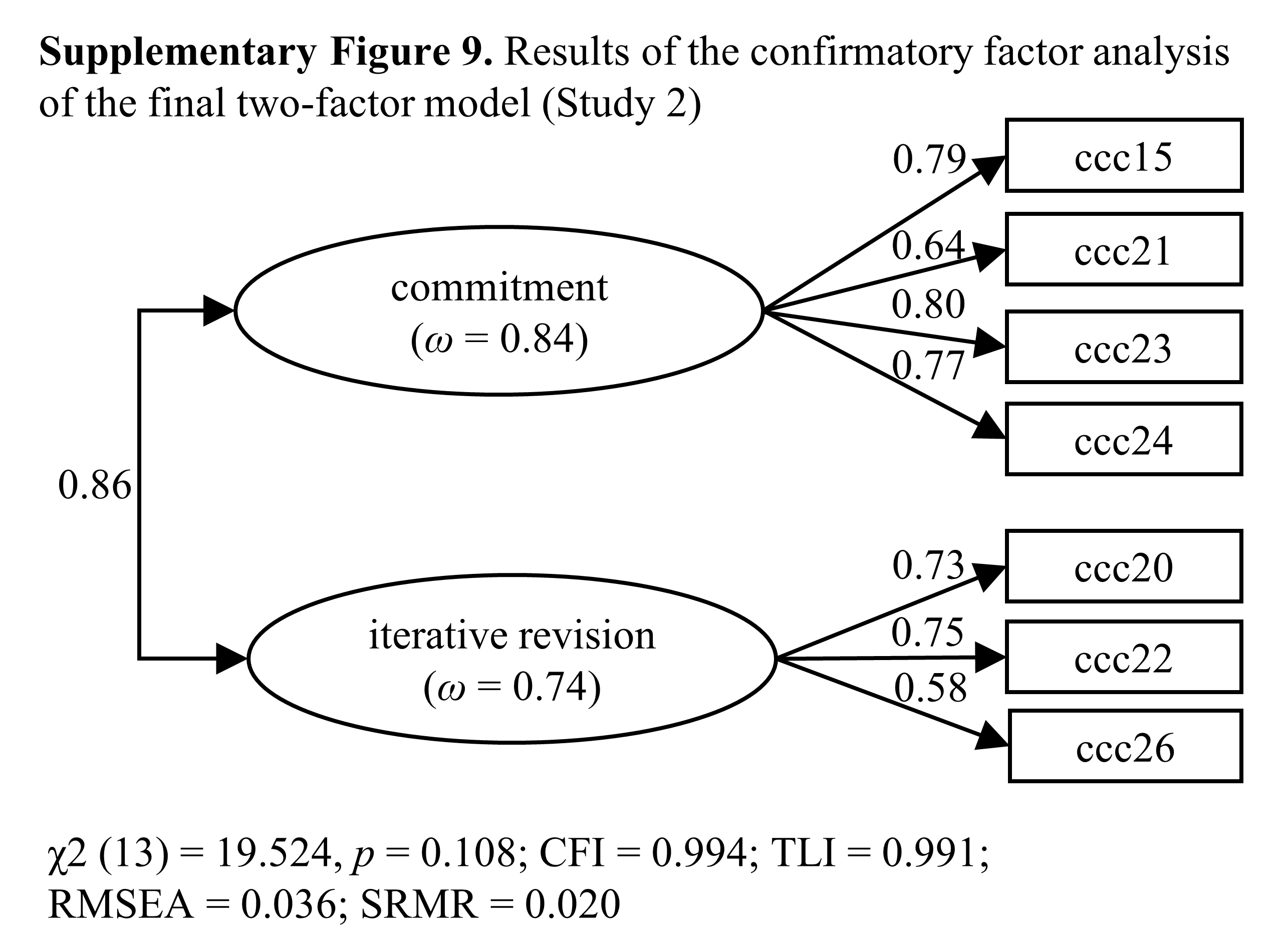

Supplement: Supplementary file 9 [file Image_9.tif]

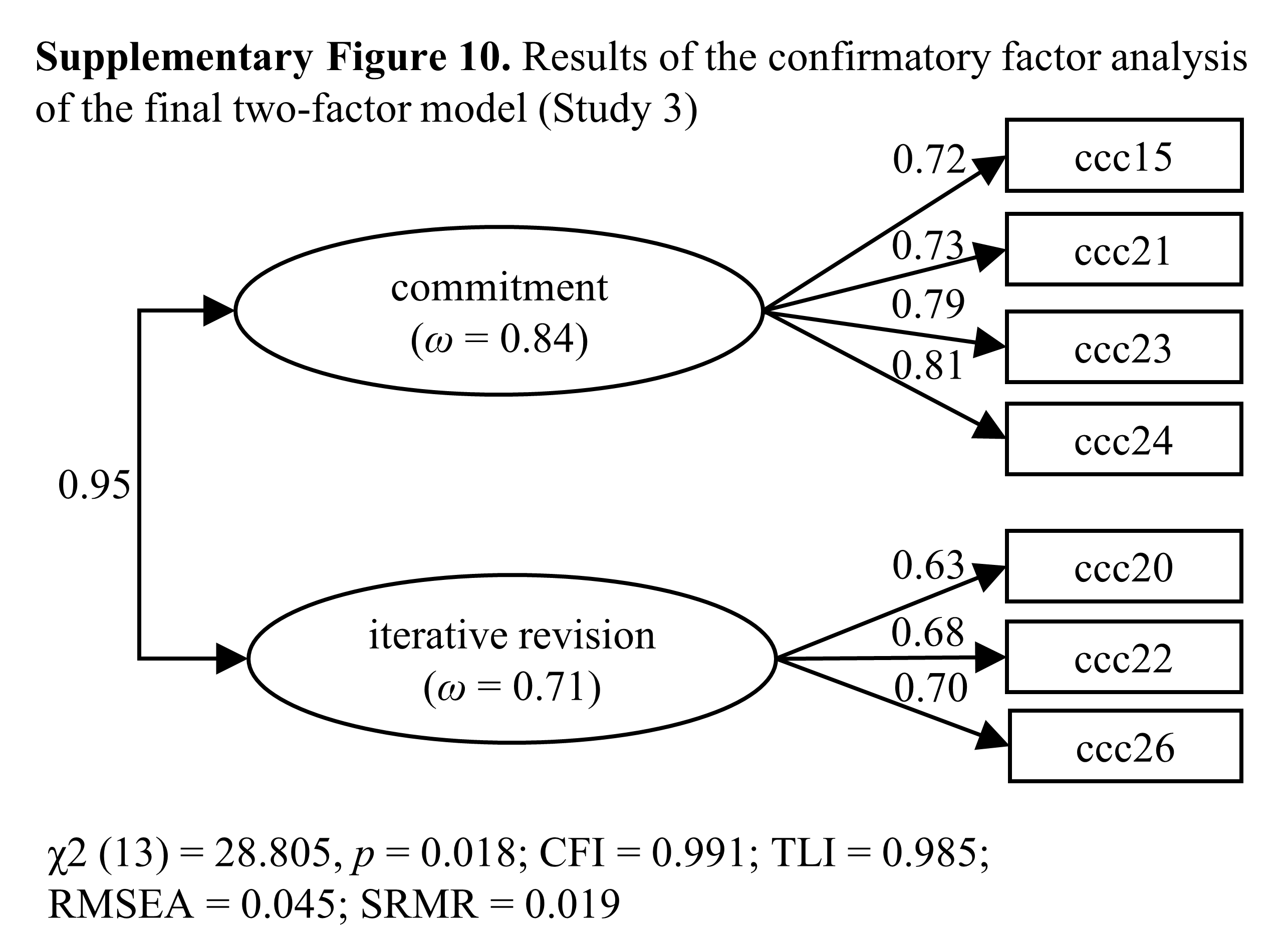

Supplement: Supplementary file 10 [file Image_10.tif]
